# Computational identification and experimental verification of a novel signature based on SARS-CoV-2–related genes for predicting prognosis, immune microenvironment and therapeutic strategies in lung adenocarcinoma patients

**DOI:** 10.3389/fimmu.2024.1366928

**Published:** 2024-03-26

**Authors:** Yuzhi Wang, Yunfei Xu, Yuqin Deng, Liqiong Yang, Dengchao Wang, Zhizhen Yang, Yi Zhang

**Affiliations:** ^1^ Department of Laboratory Medicine, Deyang People's Hospital, Deyang, Sichuan, China; ^2^ Department of Laboratory Medicine, Chengdu Women's and Children's Central Hospital, Chengdu, Sichuan, China; ^3^ Department of Cardiology, Jianyang People's Hospital, Jianyang, China; ^4^ Pathogenic Microbiology and Clinical Immunology Key Laboratory of Deyang City, Deyang People's Hospital, Deyang, Sichuan, China; ^5^ College of Medical Technology, Chengdu University of Traditional Chinese Medicine, Chengdu, Sichuan, China; ^6^ Department of Blood Transfusion, Deyang People's Hospital, Deyang, Sichuan, China

**Keywords:** lung adenocarcinoma, SARS-CoV-2, prognostic signature, machine learning, immunotherapy

## Abstract

**Background:**

Early research indicates that cancer patients are more vulnerable to adverse outcomes and mortality when infected with severe acute respiratory syndrome coronavirus 2 (SARS-CoV-2). Nonetheless, the specific attributes of SARS-CoV-2 in lung Adenocarcinoma (LUAD) have not been extensively and methodically examined.

**Methods:**

We acquired 322 SARS-CoV-2 infection-related genes (CRGs) from the Human Protein Atlas database. Using an integrative machine learning approach with 10 algorithms, we developed a SARS-CoV-2 score (Cov-2S) signature across The Cancer Genome Atlas and datasets GSE72094, GSE68465, and GSE31210. Comprehensive multi-omics analysis, including assessments of genetic mutations and copy number variations, was conducted to deepen our understanding of the prognosis signature. We also analyzed the response of different Cov-2S subgroups to immunotherapy and identified targeted drugs for these subgroups, advancing personalized medicine strategies. The expression of Cov-2S genes was confirmed through qRT-PCR, with GGH emerging as a critical gene for further functional studies to elucidate its role in LUAD.

**Results:**

Out of 34 differentially expressed CRGs identified, 16 correlated with overall survival. We utilized 10 machine learning algorithms, creating 101 combinations, and selected the RFS as the optimal algorithm for constructing a Cov-2S based on the average C-index across four cohorts. This was achieved after integrating several essential clinicopathological features and 58 established signatures. We observed significant differences in biological functions and immune cell statuses within the tumor microenvironments of high and low Cov-2S groups. Notably, patients with a lower Cov-2S showed enhanced sensitivity to immunotherapy. We also identified five potential drugs targeting Cov-2S. *In vitro* experiments revealed a significant upregulation of GGH in LUAD, and its knockdown markedly inhibited tumor cell proliferation, migration, and invasion.

**Conclusion:**

Our research has pioneered the development of a consensus Cov-2S signature by employing an innovative approach with 10 machine learning algorithms for LUAD. Cov-2S reliably forecasts the prognosis, mirrors the tumor’s local immune condition, and supports clinical decision-making in tumor therapies.

## Introduction

Lung cancer (LC) ranks as the foremost cause of cancer-related mortality globally, accounting for about 2.3 million new cases and 1.8 million deaths annually (1, 2). The outlook for patients with metastatic cancer is very grim, as only 5% of them are expected to survive for 5 years. The survival rate for patients with tumors confined to the lungs varies between 33% and 60% (3). Based on the histological categorization of LC, around 85% of LCs are non-small-cell lung cancer (NSCLC), with the majority being lung adenocarcinoma (LUAD) (4). The clinical outcome is closely linked to the early identification and diagnosis, and the failure to do so often results in missing the best chance for clinical intervention. Surgical removal is advised for individuals diagnosed with stage I or II illness. In the treatment of NSCLC for patients in advanced stages, targeted therapy and immunotherapy are alternative systemic therapeutic approaches, in addition to traditional radiotherapy and chemotherapy. The selection of these strategies is based on the gene mutation scenarios and the expression of programmed cell death protein-ligand 1 (PD-L1) (5). The molecular and phenotypic diversity of LUAD is significant, with around 60% of cases having a driver mutation that is oncogenic. This mutation is often linked to specific clinicopathological characteristics and can help predict the response to treatment (6, 7). Despite advancements in genotype-based diagnosis and therapy modalities, the survival rate continues to be poor (8). Therefore, it is crucial to investigate new dependable biomarkers for the early detection and prediction of prognosis, as well as to offer prognostic markers and therapeutic objectives for LUAD.

Severe Acute Respiratory Syndrome Coronavirus 2 (SARS-CoV-2), a highly contagious positive-sense, single-stranded RNA virus, is believed to have originated from a zoonotic source and quickly transmitted among humans via respiratory droplets and physical contact (9). Since its emergence in late 2019, the swift worldwide proliferation of SARS-CoV-2 infection has been termed the Coronavirus Disease 2019 (COVID-19) pandemic. According to epidemiological data, individuals diagnosed with cancer, especially those undergoing anticancer therapy, have a significantly increased susceptibility to SARS-CoV-2 infection. This heightened vulnerability leads to a higher occurrence of severe complications and unfavorable prognosis due to their compromised immune system (10, 11). Furthermore, numerous symptoms may persist in numerous COVID-19 patients even after the acute infection has been eliminated. The impact of the COVID-19 pandemic on cancer is closely linked to the functioning of the immune system. There is a strong connection between viruses and cancers, as evidenced by multiple studies. Viruses are responsible for causing over 15% of malignancies (12). Cell transformation and carcinogenesis triggered by viral infections, such as those caused by human papillomavirus (HPV), hepatitis B and C viruses (HBV, HCV), Epstein-Barr virus (EBV), and human T-lymphoma virus, are well-documented (13–16). In a manner akin to SARS-CoV-2, SARS-CoV-1 disrupts numerous signaling pathways linked to malignant cell transformation (17). However, the carcinogenic potential of SARS-CoV-2 is yet to be fully understood and warrants further investigation. Policard et al. highlighted a range of genes influenced by SARS-CoV-2 infection, including the E2F transcription factor and RB1, indicating a possible role of SARS-CoV-2 in tumorigenesis. The link between malignancies and SARS-CoV-2 infection remains incompletely explored. Given that viruses can influence tumor progression via specific target genes, the role of SARS-CoV-2 target genes in cancer deserves thorough examination (18).

In this research, we provided a comprehensive overview of the SARS-CoV-2 infection–related genes (CRGs) identified in LUAD. Multiple machine learning algorithms were used to develop the SARS-CoV-2 score (Cov-2S), which is a prognostic model designed to predict the OS of patients with LUAD. Furthermore, the Cov-2S has the capability to assess the landscape of the tumor’s immune micro-environment and its responsiveness to both immunotherapy and chemotherapy. The results of our study reveal the important regulatory functions of CRGs in the progression of LUAD and offer potential targets for precise treatment of LUAD.

## Materials and methods

### Data resources

Transcriptome profiling data and patient survival details for LUAD were sourced from The Cancer Genome Atlas (TCGA) database to create training sets. This dataset, TCGA-LUAD, comprised 503 cases and included clinical information, genetic mutations, and copy number variation (CNV) data. For testing purposes, expression profiles from the Gene Expression Omnibus (GEO) for the following datasets were also acquired: GSE72094 (n=398), GSE68465 (n=442), and GSE31210 (n=226). All expression data were standardized to Transcripts Per Million (TPM) format to facilitate consistent dataset comparison. Following that, the “sva” package was utilized to eliminate batch effects. A total of 322 CRGs were obtained from the previous research and human protein atlas (HPA) (https://www.proteinatlas.org/) database (19), as shown in **Supplementary Table 1**.

### Enrichment pathway exploration

Differential CRGs between adjacent normal and cancerous tissues were identified using the “limma” R package, applying criteria of an absolute fold change (FC) greater than 2 and a false discovery rate (FDR) below 0.05. For the analysis of differentially expressed genes (DEGs), Kyoto Encyclopedia of Genes and Genomes (KEGG) and Gene Ontology (GO) enrichment analyses were conducted using the R packages “clusterProfiler” and “org.Hs.eg.db”. Gene set variation analysis (GSVA) was performed by integrating the “h.all.v7.5.1.symbols.gmt” gene sets from MSigDB, available at https://www.gseamsigdb.org/gsea/msigdb/index.jsp (20). This was followed by gene set enrichment analysis (GSEA) to identify significantly enriched signaling pathways and biological processes among the different groups (21).

### Development and evaluation of a prognostic Cov-2S by machine learning

To develop a robust and precise prognostic signature for lung adenocarcinoma (LUAD), potential biomarkers underwent evaluation using ten integrative machine learning algorithms, namely, random survival forest (RSF), elastic network (Enet), Lasso (least absolute shrinkage and selection operator), Ridge, stepwise Cox, CoxBoost, partial least squares regression for Cox (plsRcox), supervised principal components (SuperPC), generalized boosted regression modeling (GBM), and survival support vector machine (survival-SVM). The process for signature generation involved several steps:

1. Prognostic biomarkers were initially identified using univariate Cox regression in the TCGA dataset.
2. Subsequently, 101 algorithm combinations were applied to these biomarkers to create predictive models. These models were developed using leave-one-out cross-validation (LOOCV) within the TCGA dataset.
3. All models were then tested using three GEO datasets.
4. The Harrell’s concordance index (C-index) was computed for each model across all TCGA and GEO datasets. The model with the highest average C-index was deemed the most effective.

Previous studies provide more in-depth information on comparable machine learning algorithms (22). Following this, LUAD patients were stratified into high and low Cov-2S groups based on the median Cov-2S score. The predictive capability of Cov-2S was assessed with a time-dependent ROC curve, generated via the R-package “time-ROC” (23). Additionally, both univariate and multivariate Cox analyses were conducted to identify risk factors among clinical characteristics and Cov-2S for LUAD prognosis.

Univariate and multivariate Cox regression analyses were conducted to investigate the potential of Cov-2S and clinical parameters as independent prognostic indicators for LUAD patients. The independent prognostic indicators were integrated into a nomogram using the R package “rms” to predict 1-, 3-, and 5-year survival rates. Calibration plots were generated to assess the alignment between the survival rates predicted by the nomogram and the observed survival rates.

### Unsupervised clustering analysis

The creation of the “ConsensusClusterPlus” R package enabled the execution of an unsupervised cluster analysis on Cov-2S mRNA expression profiles (24). The ideal number of clusters was identified by choosing the k value that minimized the sum of squares within each cluster, and the classification’s stability was verified through 1000 repetitions. Moreover, the high-latitude data dimension was decreased by employing principal component analysis (PCA) from the R package “ggplot2” to examine if the gene Cov-2S could categorize patients into clusters.

### Somatic variants analysis and copy number variation analysis

The R package “maftools” was used to present the waterfall plots of the top 20 genes with the highest mutation frequencies in the mutation landscape. The calculation of the tumor mutation burden (TMB) involves determining the overall count of non-synonymous mutations present in every individual. The interaction between gene mutations was determined by using “maftools” to identify genes with significant mutations (P< 0.05) between the different groups. Only genes that underwent mutations exceeding 30 occurrences in at least one group were taken into account in both analyses. The GISTIC (Genomic Identification of Significant Targets in Cancer) 2.0 pipeline, accessible via GenePattern at https://genepattern.broadinstitute.org/, was employed to analyze copy number variation data. This analysis identified significant regions of amplification and deletion, as well as discrete copy number statuses of all genes across different Cov-2S groups. Additionally, to assess the extent of genomic alterations, metrics such as the fraction of genome altered (FGA), fraction of genome gained (FGG), and fraction of genome lost (FGL) were calculated for each sample (25). FGA was determined by calculating the proportion of fragment base count representing genetic variation within the genome, while FGG/FGL solely concentrated on the acquisition or depletion of genetic variation within the genome.

### Tumor microenvironment

Various algorithms, such as TIP (tumor immunophenotype) tracking (26), ESTIMATE (27), TIMER (28), MCP‐counter (29) and the single sample gene set enrichment analysis (ssGSEA) algorithm (30), were utilized for TME analysis. TIP concentrated on characterizing the immune microenvironment by referencing the seven-step cancer-immunity cycle and deducing the proportions of diverse tumor-infiltrating immune cells. The ESTIMATE algorithm was employed to compute the ESTIMATE score, immune score, and stromal score, thereby assessing the tumor’s immune and stromal components. Additionally, the abundance of various immune cell types infiltrating the tumor was estimated using algorithms such as TIMER, MCP-counter, and single-sample Gene Set Enrichment Analysis (ssGSEA).

### Evaluation of immunotherapy response

The Tumor Immune Dysfunction and Exclusion (TIDE) web tool, available at http://tide.dfci.harvard.edu, was employed to predict immunotherapy responses across various hypoxia subtypes of tumors (31). Additionally, the unsupervised subclass mapping (submap) method was utilized to assess the expression similarity between lung adenocarcinoma (LUAD) patients with different hypoxia subtypes and those exhibiting varied outcomes following immunotherapy. This approach posits that greater similarity in expression profiles between patient pairs suggests more closely aligned clinical outcomes (32). Furthermore, three independent external datasets (GSE78220, NIHMS1611472, and IMvigor210) were selected to investigate the correlation between the Cov-2S score and the efficacy of immunotherapy treatments.

### Prediction of chemotherapy drug sensitivity

Cancer cell lines (CCLs) drug sensitivity data were sourced from the Cancer Therapeutics Response Portal (CTRP v2.0, available at https://portals.broadinstitute.org/ctrp) and the PRISM Repurposing dataset (PRISM, accessible at https://depmap.org/portal/prism) (2, 33). The CTRP scrutinized 481 compounds against 835 CCLs, whereas the PRISM Repurpose initiative examined 1448 compounds across 482 CCLs. Prior to further analysis, compounds with missing values (NAs) in over 20% of the samples were excluded. The ISOpure algorithm was implemented to minimize the influence of non-tumor components in the analysis (34). Additionally, the “pRRophetic” package’s built-in ridge regression model was employed to predict the area under the curve (AUC) value for each compound in individual patients. This estimation utilized a combination of the meta-set purified expression profile and the drug sensitivity data (35).

### Cell culture

Human lung adenocarcinoma cell lines A549 and H838, along with the normal bronchial epithelial cell line BEAS-2B, were acquired from Procell (Wuhan, China) and the American Type Culture Collection (Manassas, VA, USA), respectively. In a controlled environment at 37°C and 5% CO2, H838 cells were maintained in RPMI-1640 medium supplemented with 10% fetal bovine serum (FBS), while A549 and BEAS-2B cells were cultured in Dulbecco’s Modified Eagle Medium (DMEM) also containing 10% FBS. Small interfering RNA (siRNA) constructs targeting GGH were obtained from Shanghai Hanheng and introduced into A549 and H838 cells using Lipofectamine 3000 for effective GGH knockdown. The siRNA sequences were as follows: siRNA-NC, 5’- CGGGCCATGAAACGCCCATGG-3’; siRNA1-GGH, 5’-GCTGTTTAACATGGTGATTTG-3’; siRNA2-GGH, 5’-GGGACCCACTGAGGTAGTTAA-3’. After 48 hours, the effectiveness of knockdown was assessed through immunoblotting, and the cells were then utilized for additional experiments.

### Cell proliferation assay

The viability of cells was assessed using Cell Counting Kit-8 (CCK-8) and colony formation experiments. In the CCK-8 trial, 1000 cells were planted in 96-well dishes and incubated for 24, 48, and 72 hours. Subsequently, 10 µl of CCK-8 reagent (Saiku, Shanghai, China) was added to each well containing 100 µl of culture medium, and the plates were incubated at 37°C. After 2 hours, the optical density (OD) at 450 nm was measured using a micro-plate reader. In the colony formation assay, 500 cells subjected to treatment were plated in each well of 12-well plates. After an incubation period of 10 days, the plates were washed twice with phosphate-buffered saline (PBS). The cells were then fixed with 4% paraformaldehyde for 30 minutes and stained using a crystal violet staining solution for 10 minutes. Finally, they were stained with a solution of crystal violet for 10 minutes.

### Cell migration and invasion assay

For the wound healing experiment, 5x10^5 cells were seeded into each well of a 6-well plate. The following day, a sterile 20 μl pipette tip was used to create a scratch (wound) on the cell surface. Post-wounding, non-adherent cells were washed off with phosphate-buffered saline (PBS) and replaced with fresh serum-free medium for a 24-hour period. The progression of wound closure was monitored and assessed using a light microscope. For cell migration assessment, Transwell chambers (24-well, BIOFIL, China) were employed. The lower chamber was filled with 0.6 ml of Dulbecco’s Modified Eagle Medium (DMEM) supplemented with 20% fetal bovine serum (FBS). Meanwhile, approximately 7x10^4 cells, resuspended in basic medium, were added to the upper chamber and incubated overnight at 37°C in a 5% CO2 atmosphere. After 24 hours, the cells were fixed with 4% paraformaldehyde and stained with 2.5% crystal violet for subsequent analysis. ImageJ was used to randomly select and count three microscopic views. Cell invasion assay also employed Transwell chambers. 70 µl of diluted Matrigel was applied to the upper chamber prior to coating. The migration was performed without coating matrigel surface. The following procedures are comparable to the migration process.

### Cell viability assay

Cell viability was determined by CCK8 cytotoxicity assay. Briefly, cells were seeded in 96-well plates at 5000 cells/well and treated with at 37°C with Ispinesib (0, 30, 60, 120 or 240 nM, MedChem Express, Monmouth Junction, NJ, USA, Cat No.HY-50759), Paclitaxel (0, 10, 20, 40 or 80 nM, MedChem Express, Monmouth Junction, NJ, USA, Cat No. HY-B0015) and Epothilone-b (0, 40, 80, 160 or 320 nM, MedChem Express, Monmouth Junction, NJ, USA, Cat No. HY-17029) for 24 h, respectively. Cells were incubated with CCK8 (10 μl/well) for 2 h at 37°C and then spectrophotometrically quantified at 450 nm.

### Quantitative real-time PCR and immunohistochemistry

The total RNA was extracted with RNA isolation Kit (AG, Changsha, China) according to the product protocol. The extracted RNA underwent reverse transcription using the Reverse Transcription Kit (Promega, Madison, Wisconsin) to synthesize complementary DNA (cDNA), setting the stage for quantitative real-time PCR (qRT-PCR). The qRT-PCR analysis was performed on the Roche 480II quantitative real-time gene amplification instrument (Roche, Oregon, USA), utilizing SYBR Premix Ex Taq II (Promega, Wisconsin, USA). For normalization and control purposes, GAPDH was employed as an endogenous reference. Relative gene expression levels were calculated and analyzed using the 2^-ΔΔCt^ method. The specific primers used in this study are detailed in **Supplementary Table 2**. The LUAD tissue microarray (HLugA020PG02) was acquired from Outdo Biotech, and its ethics approval was obtained. The IHC procedure was performed according to the previously mentioned protocol (36). The main antibodies used were anti-GGH (Huaan, Hangzhou, HA721359, 1:100). In this study, the immunostaining intensity was scored using the following scale: 0 for negative staining, 1 for light yellow staining, 2 for brownish yellow, and 3 for tan. Additionally, the extent of the immunostaining area was evaluated as: 1 for less than one-third coverage, 2 for coverage between one-third and two-thirds, and 3 for more than two-thirds coverage. The final score for biomarker expression was determined by multiplying the immunostaining intensity score by the immunostaining area score.

### Statistical analysis

All statistical analyses in this study were conducted using R software 4.1 and GrapdPad Prism 5. For categorical data, Chi-square tests and Fisher’s exact tests were employed. The Wilcoxon signed-rank test was utilized for comparing paired continuous variables. Pearson correlation analysis was applied to assess correlations between continuous variables. Statistical significance levels were defined as follows: extremely significant (****) for p < 0.0001, highly significant (***) for p < 0.001, significant (**) for p < 0.01, marginally significant (*) for p < 0.05, and not significant (ns) for p ≥ 0.05.

## Results

### Transcriptional and genetic alterations of CRGs in LUAD patients

**Figure 1:**
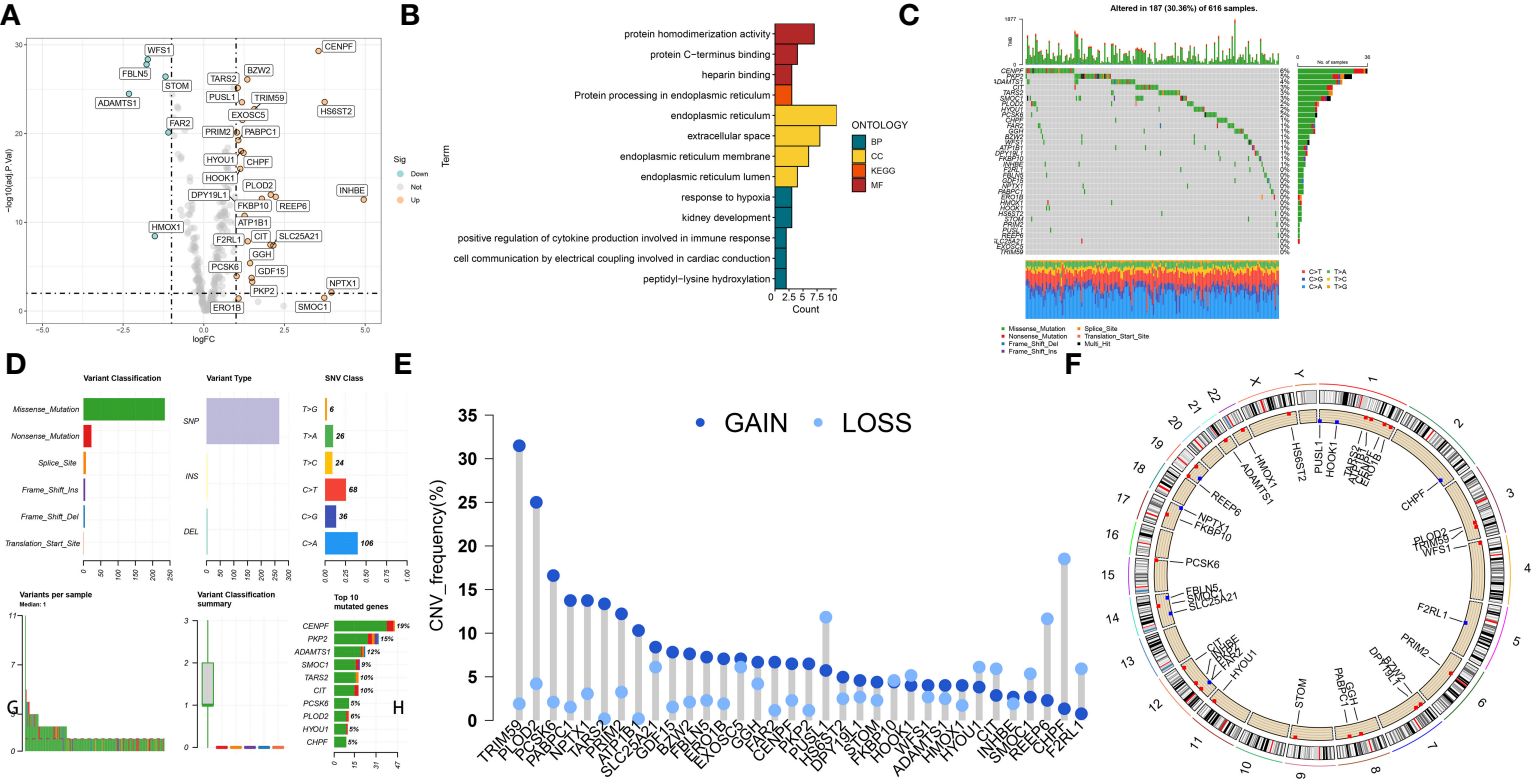
The landscape of SARS-CoV-2 infection–related proteins (CRGs) in TCGA-LUAD set. **(A)** Volcano plot of the DECRGs. **(B)** GO categories [molecular function (MF), biological process (BP) and cellular component (CC)] and KEGG pathway analysis for DECRGs. **(C, D)** The mutation summary and details of DECRGs in the LUAD patients. **(E)** CNV mutation situation of the DEPCDRGs. **(F)** The location of CNV alterations of DECRGs on chromosomes.

The CRGs expression patterns between adjacent and cancerous tissues were compared in the TCGA LUAD and identified 34 differently expressed SARS-CoV-2 infection-related genes (DECRGs) containing 28 upregulated and 6 downregulated genes (**Figure 1A**). We conducted GO and KEGG enrichment analysis for DECRGs to determine the functions and pathways that had the greatest level of involvement. In terms of GO analysis, the DECRGs were mainly enriched in “protein homodimerization activity”, “endoplasmic reticulum” and “response to hypoxia” (**Figure 1B**). In the KEGG pathway, the DECRGs showed significant enrichment in the category of “protein processing in the endoplasmic reticulum” (**Figure 1B**). In order to delve deeper into the correlation between genomic changes and expressions of DECRGs in LUAD, the TCGA-LUAD project compiled the genomic modifications of these genes. Analysis of somatic mutations revealed that DECRGs mutations were present in 187 out of 616 samples (30.36%). Among these, CENPF (6%), PKP2 (5%) and ADAMTS1 (4%) exhibited the highest gene mutation rates (**Figure 1C**). The predominant mutation types in these genes are nonsense mutations (**Figure 1D**). The findings presented in **Figure 1E** demonstrate a relatively low frequency of CNVs among the DECRGs. Notably, genes such as TRIM59, PLOD2, PCSK6, PABPC1, and NPTX1 exhibited amplifications in their copy numbers. In contrast, genes like SMOC1, REEP6, CHPF, and F2RL1 showed deletions in their copy numbers. **Figure 1F** depicts the chromosomal locations of these DECRGs in LUAD patients. Additionally, among these DECRGs, 16 genes were identified as having significant correlations with the prognosis of LUAD patients.

### Construction of prognostic Cov-2S by integrative machine learning algorithms

**Figure 2:**
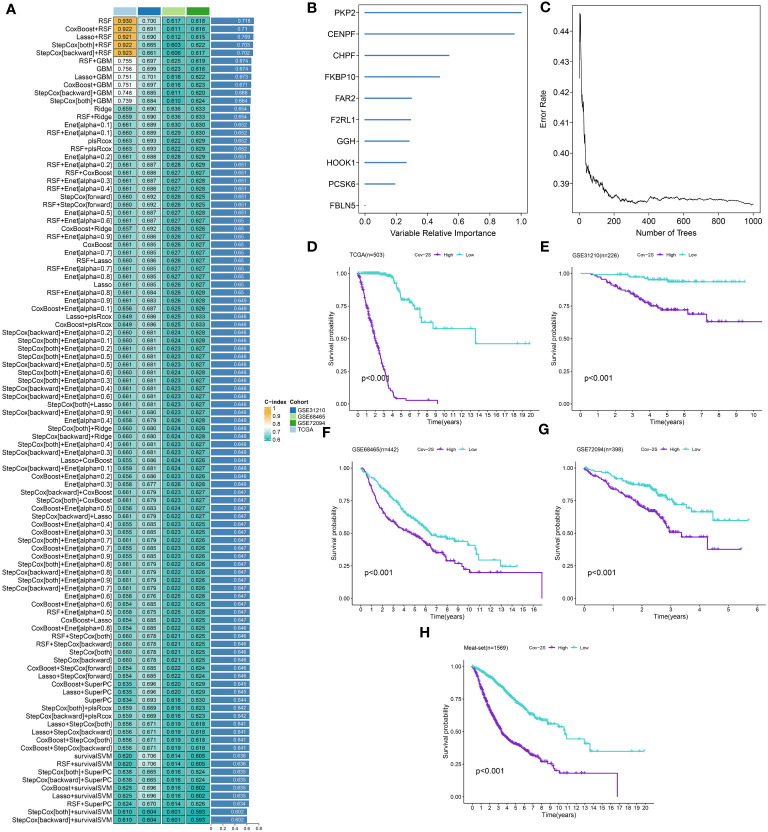
A SARS-CoV-2 score (Cov-2S) was established and validated via the machine learning-based integrative procedure. **(A)** A total of 101 kinds of machine learning algorithms were used to obtain the optimal model and calculated the C-index of each model for all sets. **(B, C)** The number of trees for determining the Cov-2S with minimal error and the importance of the 10 CRGs based on the RSF algorithm. **(D-H)** Kaplan–Meier curves of OS according to the Cov-2S in TCGA, GSE31210, GSE68465, GSE72094 and meta-set.

After identifying 16 potential prognostic genes, a machine learning-based integrative approach was employed to create a reliable and consistent prognostic model. In total, 101 different types of prognostic models based on machine learning were acquired, and their C-index for both the training and testing sets were displayed in **Figure 2A** and **Supplementary Table 3**. The RFS framework exhibits a highest average C-index of 0.716 was suggested as the optimal model (**Figure 2A**, **Supplementary Table 4**). The Cov-2S was determined for every individual in all groups by analyzing the expression levels of 10 genes included in the model (**Figures 2B, C**). LUAD cases were divided into high and low Cov-2S groups based on the median value of Cov-2S. As anticipated, patients with low Cov-2S in LUAD exhibited a higher OS rate in the training, testing, and meta sets (**Figures 2D-H**). In addition, the discriminative ability of Cov-2S group for the prognosis of LUAD patients is not influenced by clinical feature subtypes (**Supplementary Figure 1**).

### Evaluation of the Cov-2S signature

**Figure 3:**
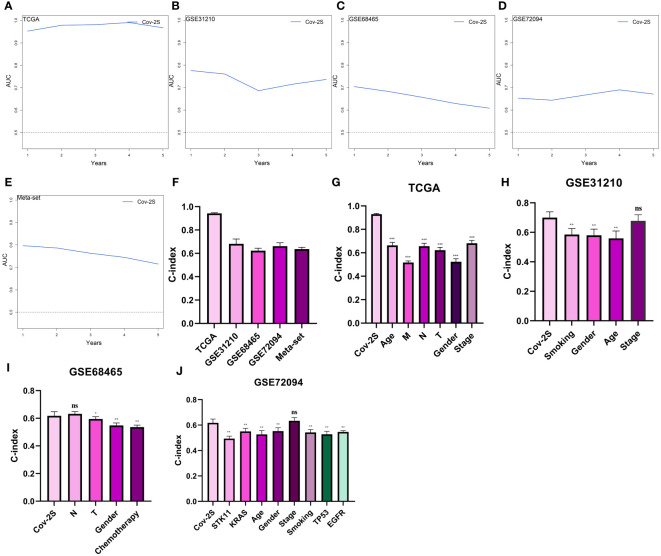
Evaluation of the Cov-2S. **(A-E)** Time-dependent receiver operating characteristic curve of Cov-2S for predicting the prognosis of LUAD patients from TCGA, GSE31210, GSE68465, GSE72094 and meta-set. **(F)** The C-index of the Cov-2S for the TCGA, GSE31210, GSE68465, GSE72094 sets. **(G-J)** The C-index of the Cov-2S and other clinical factors in the TCGA, GSE31210, GSE68465, GSE72094 sets. ns, not significant. *P < 0.05, **P < 0.01, ***P < 0.001.

**Figure 4:**
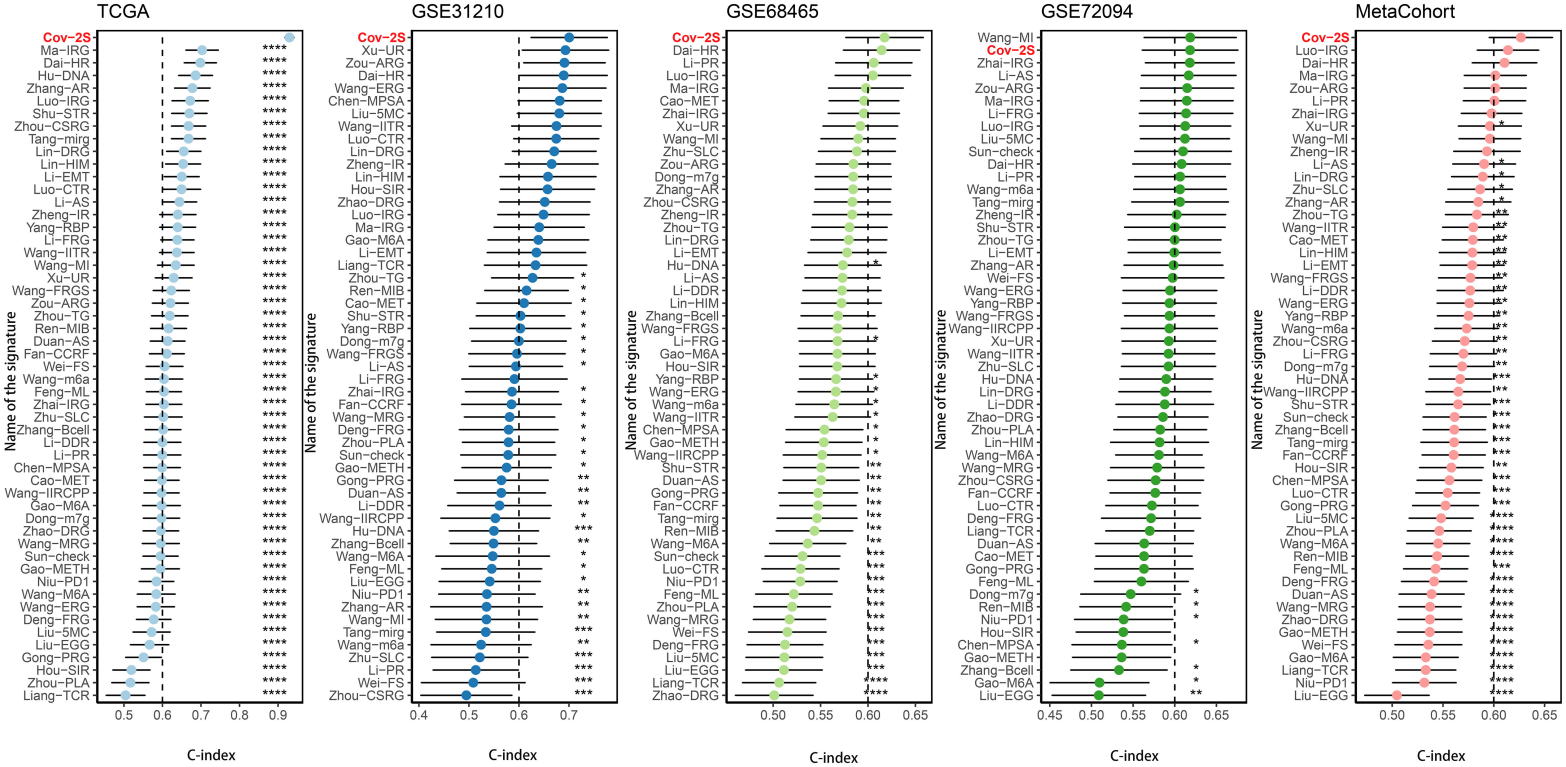
Comparison of Cov-2S and other gene expression-based prognostic signatures in LUAD based on the TCGA, GSE31210, GSE68465, GSE72094 and meta-set. ns, not significant. *P < 0.05, **P < 0.01, ***P < 0.001, **** P < 0.0001.

The discrimination of Cov-2S was assessed using Time-ROC analysis, yielding AUC values ranging from 0.94 to 0.98 in TCGA, 0.68 to 0.78 in GSE31210, 0.61 to 0.72 in GSE98465, 0.65 to 0.68 in GSE72094, and 0.72 to 0.9 in the meta-set (**Figures 3A-E**). Similar to the Time-ROC curve results, the C-index range for all sets is 0.62-0.93, with the TCGA set having the highest C-index (**Figure 3F**). The Chi-square test results indicated that individuals with advanced T, N, M stages and deceased status exhibited elevated Cov-2S levels (**Supplementary Figure 2**). Additionally, the prognostic prediction of Cov-2S was compared with other clinical and molecular variables. As displayed in **Figures 3G-J**, Cov-2S had distinctly superior accuracy than almost all clinicopathological measures including M, T, T, gender, M and TP53, KRAS, or EGFR mutations. In order to investigate if Cov-2S functioned as a standalone prognostic indicator, univariate and multivariate cox analyses were conducted by including various clinicopathological traits and Cov-2S. The results suggested the Cov-2S as a reliable prognostic indicator for LUAD patients independently (**Tables 1**–**4**). Incorporating independent predictors into the nomogram construction, we observed that Cov-2S exerted the most significant influence on survival prediction (**Supplementary Figures 3A-D**). Evaluation of the prediction accuracy of the model using calibration curves revealed close alignment between the predicted calibration curves at the 1-, 3-, and 5-year calibration points and the standard curve (**Supplementary Figures 3E-H**). Numerous predictive patterns have been developed for LUAD. In order to assess the predictive efficacy of Cov-2S in relation to other prognostic indicators, a random selection of 58 constructed prognostic indicators for LUAD were gathered and their C-index was computed. As shown in **Figure 4**, the C-index of our Cov-2S exceeded that of the majority of these prognostic signatures across all analyzed datasets.

**Table 1:** Univariate and multivariate Cox analysis of the clinicopathological features and Cov-2S with OS for TCGA cohort.

	Univariate Cox		Multivariate Cox	
Characteristics	HR(95%CI)	*P* value	HR(95%CI)	*P* value
Stage	1.977(1.586-2.463)	**< 0.001**	1.487(1.049-2.109)	**0.026**
N	1.942(1.575-2.394)	**< 0.001**	1.251(0.93-1.681)	0.139
T	1.816(1.386-2.38)	**< 0.001**	1.423(1.034-1.958)	**0.03**
Age	1.038(0.822-1.31)	0.754	NA	NA
Sex	1.041(0.847-1.28)	0.7	NA	NA
M	1.727(1.18-2.527)	**0.005**	0.756(0.479-1.191)	0.227
Cov-2S	0.106(0.075-0.149)	**< 0.001**	0.127(0.086-0.187)	**< 0.001**

Significant value is given in bold.

**Table 2:** Univariate and multivariate Cox analysis of the clinicopathological features and Cov-2S with OS for GSE68465 cohort.

	Univariate Cox		Multivariate Cox	
Characteristics	HR(95%CI)	*P* value	HR(95%CI)	*P* value
N	2.029(1.689-2.438)	**< 0.001**	1.947(1.603-2.366)	**< 0.001**
T	2.062(1.587-2.68)	**< 0.001**	1.797(1.36-2.374)	**< 0.001**
Gender	1.262(1.051-1.516)	**0.013**	1.267(1.044-1.539)	**0.017**
chemotherapy	1.412(1.15-1.734)	**< 0.001**	1.246(1.004-1.545)	**0.046**
Cov-2S	0.728(0.607-0.875)	**< 0.001**	0.784(0.648-0.948)	**0.012**

Significant value is given in bold.

**Table 3:** Univariate and multivariate Cox analysis of the clinicopathological features and Cov-2S with OS for GSE31210 cohort.

	Univariate Cox		Multivariate Cox	
Characteristics	HR(95%CI)	*P* value	HR(95%CI)	*P* value
smoking	1.417(0.882-2.277)	0.15	NA	NA
gender	1.344(0.839-2.152)	0.219	NA	NA
age	1.263(0.777-2.052)	0.346	NA	NA
stage	2.774(1.732-4.441)	**< 0.001**	2.015(1.233-3.293)	**0.005**
Cov-2S	0.286(0.153-0.532)	**< 0.001**	0.356(0.186-0.682)	**0.002**

Significant value is given in bold.

**Table 4:** Univariate and multivariate Cox analysis of the clinicopathological features and Cov-2S with OS for GSE72094 cohort.

	Univariate Cox		Multivariate Cox	
Characteristics	HR(95%CI)	*P* value	HR(95%CI)	*P* value
STK11	1.028(0.72-1.469)	0.879	NA	NA
KRAS	0.767(0.588-0.999)	**0.049**	0.907(0.69-1.192)	0.484
Age	1.258(0.836-1.894)	0.27	NA	NA
Gender	0.733(0.564-0.952)	**0.02**	0.725(0.553-0.95)	**0.02**
Stage	1.969(1.477-2.625)	**< 0.001**	1.921(1.43-2.58)	**< 0.001**
Smoking	1.248(0.694-2.245)	0.459	NA	NA
TP53	0.861(0.645-1.151)	0.313	NA	NA
EGFR	2.58(1.274-5.226)	**0.008**	2.147(1.047-4.4)	**0.037**
Cov-2S	0.575(0.438-0.755)	**< 0.001**	0.64(0.483-0.847)	**0.002**

Significant value is given in bold.

### Generation of Cov-2S modification patterns

**Figure 5:**
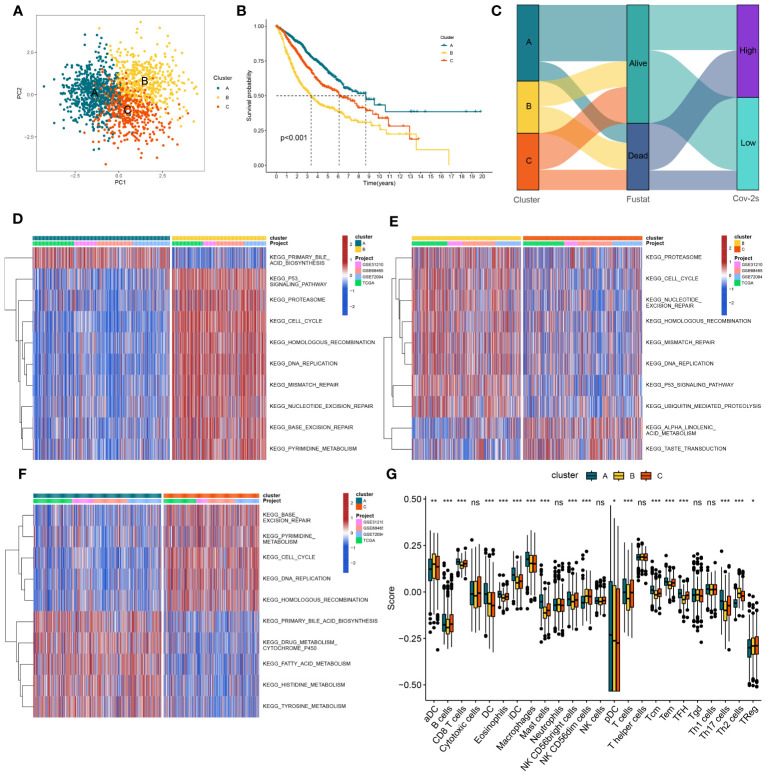
The construction of clusters based on Cov-2S. **(A)** PCA plot of three clusters. **(B)** Kaplan-Meier survival analysis between three clusters. **(C)** Alluvial diagram of clusters distributions in groups with different Cov-2S and survival outcomes. **(D-F)** GSVA analysis indicating significant enrichment of pathways in the three clusters. **(G)** The proportion of 24 kinds of immune cells in three clusters. ns, not significant. *P < 0.05, **P < 0.01, ***P < 0.001.

In order to gain a deeper understanding of the impacts of Cov-2S on LUAD, a consensus clustering analysis was conducted to categorize LUAD patients based on various alteration patterns. The heatmap of the consensus matrices and cumulative distribution function (CDF) curve (**Supplementary Figure 4**) identified that there were two clusters (cluster A, cluster B and cluster C) which were considered optimal. The PCA analysis confirmed a strong distribution between different groups based on the expression profiles of the Cov-2S (**Figure 5A**). Significantly, the analysis of survival demonstrated a significant difference in survival rates between the three clusters, where cluster A showed a more positive outcome and cluster B had a poorer prognosis (**Figure 5B**). **Figure 5C** illustrates the proportion of surviving and dying patients in three cluster and two Cov-2S groups. In cluster A, GSVA revealed prominent activation of various metabolic biological processes, while cluster B and cluster C were markedly enriched in various cell proliferation pathways (**Figures 5D-F**). Moreover, the ssGSEA method detected obvious disparities in the immune scores of the three distinct clusters (**Figure 5G**).

### Somatic mutation and CNV status between the different groups

**Figure 6:**
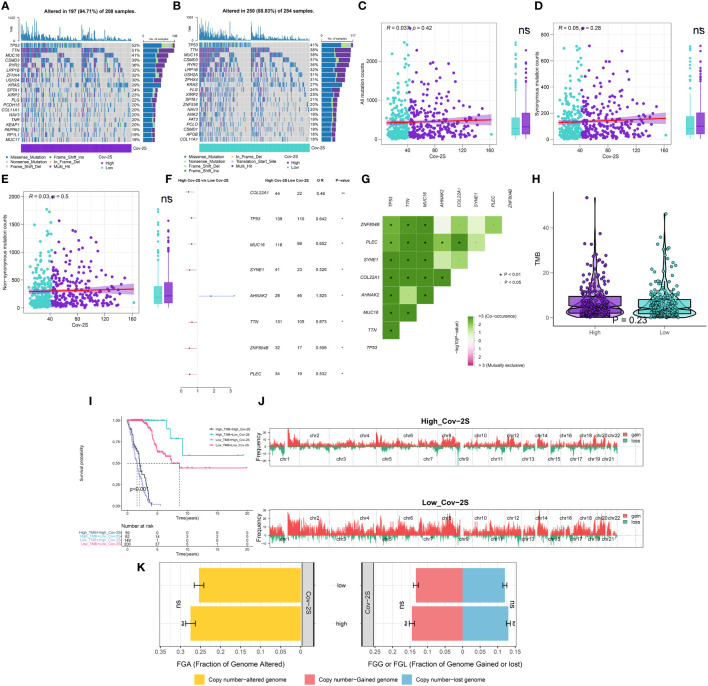
Integrated comparisons of somatic mutation and CNVs between high and low Cov-2S groups in the TCGA set. **(A, B)** Waterfall plots showing the mutation information of the top 20 genes with the highest mutation frequency in the Cov-2S groups. **(C-E)** Association between all mutation counts, synonymous mutation counts, nonsynonymous mutation counts, and Cov-2S and their distribution in the Cov-2S groups. **(F)** Differentially mutated genes between high and low Cov-2S groups are displayed as a forest plot. **(G)** Interaction effect of genes mutating differentially in patients in the Cov-2S groups. **(H)** Distribution of TMB in the Cov-2S groups. **(I)** Kaplan–Meier curves for patients stratified by both TMB and Cov-2S. **(J)** Gene fragments profiles with amplification (red) and deletion (green) among the Cov-2S groups. **(K)** Comparison of the fraction of the genome altered, lost, and gained between the Cov-2S groups. ns, not significant. * P < 0.05.

Our analyses suggested a specific somatic mutation distribution among two Cov-2S groups. As shown in **Figures 6A, B**, TP53, TTN, MUC16, CSMD3, RYR2, and LRP1B were commonly mutated in both the high and low Cov-2S groups. Somatic mutation profiles indicated that synonymous and non-synonymous mutations, as well as overall mutation counts, did not display any noteworthy variances between the high and low Cov-2S groups. However, 8 genes demonstrated significantly distinct mutation frequencies between the two groups, along with a notable presence of co-mutations (**Figures 6C-G**). Moreover, there was no statistically different variation in TMB between the high and low Cov-2S groups (**Figure 6H**). Additionally, the study revealed the correlation between TMB, Cov-2S, and prognosis, indicating that patients with low TMB and high Cov-2S experience the most unfavorable prognosis (**Figure 6I**). Following that, CNV analysis revealed distinct chromosomal alteration patterns between the high and low Cov-2S groups (**Figure 6J**). Regrettably, there were no statistical difference in genomic loss, gain, and alteration between the two Cov-2S groups (**Figure 6K**).

### TME and molecular characteristics of the Cov-2S

**Figure 7:**
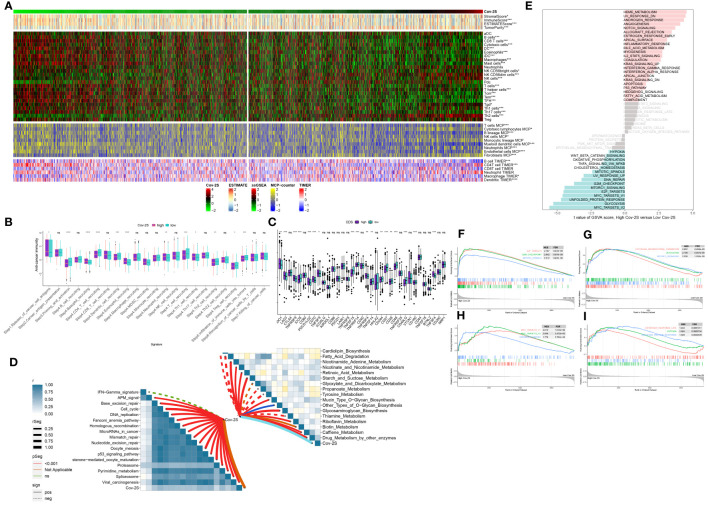
Immune-related characteristics of the Cov-2S. **(A)** Heatmap displaying the correlation between the Cov-2S and immune infiltrating cells in the meta-set. **(B)** Boxplot showing the differences of anti-cancer immunity score between Cov-2S groups. **(C)** Comparison of immune checkpoint-related genes levels between Cov-2S groups in the meta-set. **(D)** The correlations between the Cov-2S and immune-related pathways, metabolic pathways based on GSVA of GO and KEGG terms were displayed in butterfly plot. **(E)** The difference in the hallmark gene sets between different Cov-2S groups. **(F-I)** The GSEA results for the 12 overlapping upregulated hallmark pathways in terms of the high Cov-2S groups. ns, not significant. *P < 0.05, **P < 0.01, ***P < 0.001, **** P < 0.0001.

The immune condition of the TME impacts the outcome of cancer cells and anticipates responsiveness to immune checkpoint inhibitors (ICIs). Initially, we examined the correlation between Cov-2S and the infiltration of immune cells. As depicted in the illustration, the majority of individuals in the low Cov-2S group exhibited an elevated quantity of immune infiltrating cells (**Figure 7A**). Analyzing the correlation between the Cov-2S signature and the functions of the anticancer immunity cycles helps in understanding the status of anticancer immunity. Patients in the low Cov-2S group exhibited marked upregulation of various processes, including priming, activation, recruitment of CD4 T cells, and infiltration of immune cells into the tumor (**Figure 7B**). The findings indicated that patients with a low Cov-2S displayed a comparatively elevated expression of the majority of immune checkpoint genes (**Figure 7C**). Moreover, the Cov-2S exhibited a positive correlation with pathways associated with immunotherapy response and various metabolic pathways, such as the IFN-γ signature, DNA repair, nicotinamide adenine metabolism, and biotin metabolism (**Figure 7D**). GSVA was conducted to explore the underlying cancer mechanism of the Cov-2S.The findings indicated that individuals with elevated Cov-2S levels exhibited an abundance of pathways associated with cancer, including the EMT, proliferation, metabolites, and DNA repair (**Figure 7E**). Interestingly, these pathway results were confirmed in the GSEA analysis (**Figures 7F-I**).

### Sensitivity prediction of different groups to immunotherapy and chemotherapy

**Figure 8:**
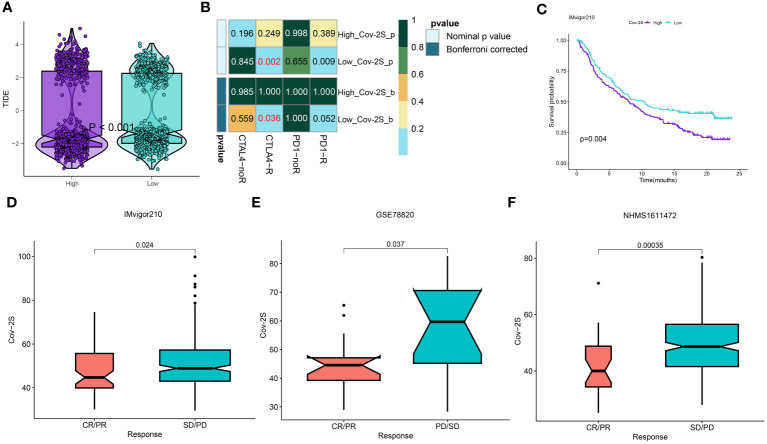
Differential putative immunotherapy response for patients from high and low Cov-2S groups. **(A)** Violin plot showing different TIDE scores from patients with different Cov-2S. **(B)** Submap analysis of the meta-set and melanoma patients with detailed immunotherapeutic information. **(C)** Kaplan-Meier curve for patients in high and low Cov-2S groups in the IMvigor210 set. **(D-F)** Box plot showing different Cov-2S from patients with immunotherapy responses in the IMvigor210, GSE78820, NHMS1611472 sets.

**Figure 9:**
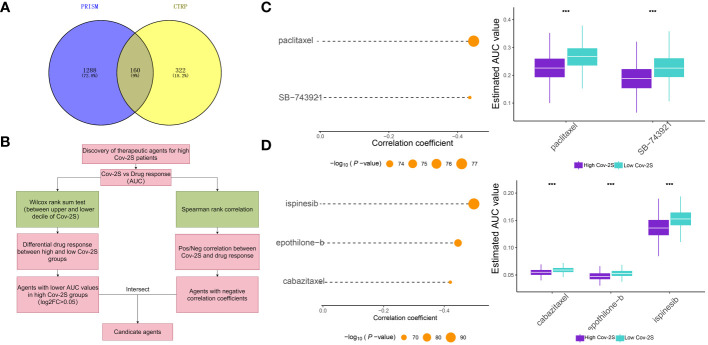
Identification of candidate drugs for high Cov-2S patients. **(A)** Data for drug prediction were sourced from the CTRP and PRISM databases. The Venn diagram illustrates the compounds contained within each database. **(B)** The process involved in exploring the drug databases of CTRP and PRISM is depicted in the flowchart. This exploration primarily encompassed the utilization of the Wilcoxon rank sum and Spearman correlation statistical algorithms. **(C)** Spearman’s correlation analysis and the analysis of differential drug response were conducted for two compounds derived from CTRP data. **(D)** Spearman’s correlation analysis and the analysis of differential drug response were conducted for three compounds derived from PRISM data. ***P < 0.001.

The treatment of LUAD has demonstrated a promising potential for the application of immunotherapy. Through TIDE and submap analysis, we assessed the immunotherapy response of two Cov-2S groups. The results showed that individuals with low Cov-2S have a decreased TIDE score, suggesting that these patients are more prone to receiving advantages from immunotherapy (**Figure 8A**). Furthermore, the Submap analysis module revealed that patients with low Cov-2S exhibit similarities to melanoma patients who responded favorably to anti-PD-1 treatment (**Figure 8B**). Given the potential of Cov-2S to accurately forecast the efficacy of immunotherapy for LUAD, we aim to investigate its ability to predict the response to ICIs in groups of patients undergoing immunotherapy. In the IMvigor210 dataset, survival analysis revealed that a high Cov-2S score was associated with a poorer prognosis compared to a low Cov-2S score (**Figure 8C**). Additionally, the complete response/partial response (CR/PR) group exhibited a lower levels of Cov-2S than the stable disease/progressive disease (SD/PD) group, as demonstrated in **Figure 8D**. In the GSE78220 and NIHMS1611472 cohorts, we also discovered that decreased Cov-2S is associated to positive response to ICIs treatment (**Figures 8E, F**). Overall, these findings suggest that Cov-2S may be linked to immunotherapy response. To identify potential drugs with the desired properties, we conducted drug response prediction separately using drug response data derived from CTRP and PRISM. CTRP and PRISM datasets encompass gene expression profiles and drug sensitivity profiles of numerous CCLs, offering a foundation for constructing drug response prediction models. A subset of 160 compounds was common to both datasets, resulting in a cumulative count of 1770 unique compounds across the combined datasets after eliminating redundancies (**Figures 9A, B**). During the cross-validation of the two pharmacogenomics databases, we identified four drugs or compounds (paclitaxel, SB-743921, cabazitaxel, epothilone-b, and ispinesib) that show promising therapeutic potential for patients with high Cov-2S. These drugs have lower estimated AUC values and exhibit a negative correlation with Cov-2S, as depicted in **Figures 9C, D**.

### Predictive efficacy of Cov-2S from a pan-cancer perspective

**Figure 10:**
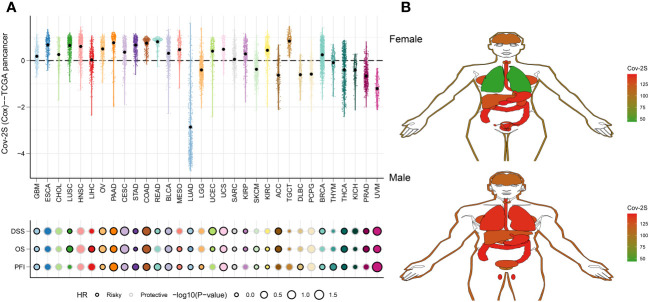
Predictive accuracy of the Cov-2S in the TCGA-pancancer set. **(A)** Distribution and predictive value of Cov-2S in solid tumors in the TCGA-pancancer set. **(B)** Differences in the distribution of Cov-2S in tumor tissues in different organs.

To assess the generalizability of Cov-2S application in different solid tumors, we constructed Cov-2S in the TCGA pan-cancer set and evaluated the distribution and predictive efficacy of Cov-2S. Our results showed a significant distribution of Cov-2S in most solid tumors, with the highest evaluated Cov-2S being in rectum adenocarcinoma and testicular germ cell tumors (**Figure 10A**). In addition, Cov-2S can be a significant risk factor for glioma, ovarian cancer, cervical squamous epithelial cell cancer, pancreatic cancer, colon cancer, bladder cancer, uterine carcinosarcoma, sarcoma, thyroid carcinoma and uveal melanoma (**Figure 10A**). Finally, we evaluated the differential expression of Cov-2S in tumor tissues in different organs. Cov-2S exhibits significant elevation in organs such as the esophagus, stomach, colon, gallbladder, and uterus in female cancer patients; while Cov-2S exhibits significant elevation in organs such as the lungs, esophagus, stomach, colon, gallbladder, and testes in male cancer patients (**Figure 10B**).

### GGH in LUAD progression

**Figure 11:**
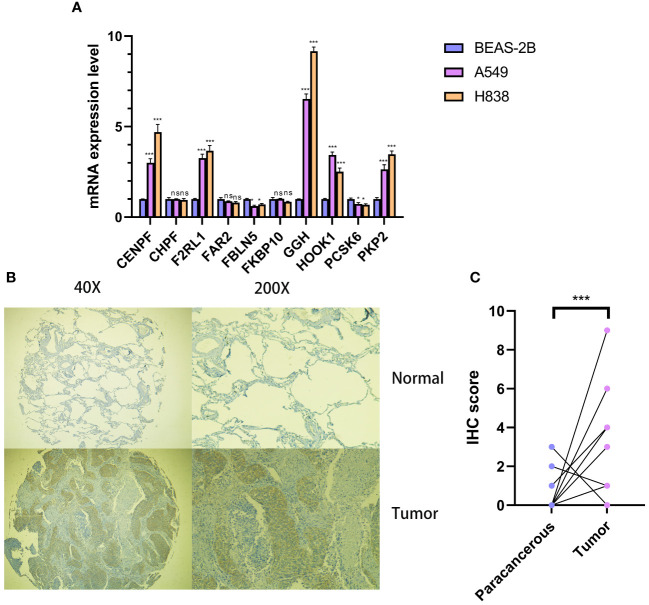
Cellular and histological and validation candidate gene expression changes. **(A)** Cov-2S genes expression in cancer and normal cell lines. beta-actin was used as the internal reference gene and experiment was performed in triplicate and at least three times. **(B, C)** IHC analysis of GGH in 10 LUAD and 10 adjacent tissues. ns, not significant. *P < 0.05, ***P < 0.001.

**Figure 12:**
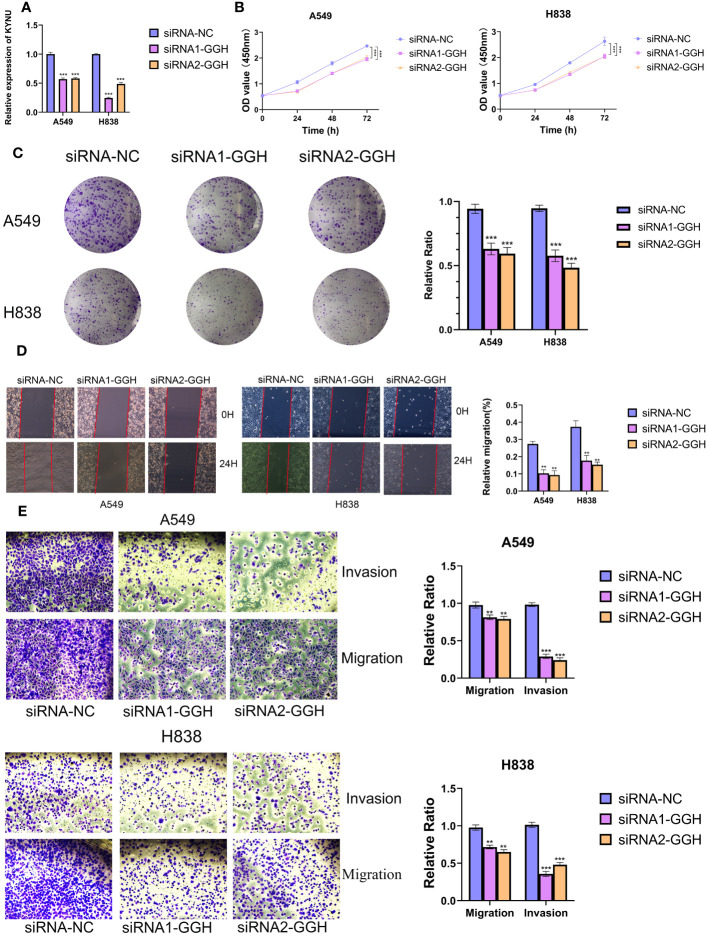
GGH promoted proliferation, migration, invasion and inhibited apoptosis of LUAD cell lines. **(A, B)** Knockdown of GGH was confirmed by qRT-PCR. **(C, D)** CCK8 and clone formation assays were performed to assess cell viability and proliferation of A549 and H838 cells. **(E)** Transwell assay was performed to assess cell migration and invasion of A549 and H838 cells. **P < 0.01, ***P < 0.001.

In order to examine the function of Cov-2S in cellular processes, we performed a comparative analysis of relative expression. The expression levels of CENPF, F2RL1, GGH, HOOK1 and PK92 were significantly elevated in LUAD cell lines by qRT-PCR experiment, while FBLN5, PCSK6 were significantly depressed in in LUAD cell lines (**Figure 11A**). IHC experiments further validated the high expression of GGH protein in LUAD tissues (**Figures 11B, C**). Given that GGH exhibits the most prominent upregulation in LUAD cells, and there is a lack of prior research on its involvement in LUAD, we have opted to investigate GGH in subsequent experiments. Successful GGH knockdown were detected by qRT-PCR (**Figure 12A**). The CCK-8 test showed a markedly diminished in cellular growth capacity after transfection with GGH siRNA (**Figure 12B**). Transfection with GGH siRNA resulted in a significant reduction in the number of colonies formed, as demonstrated by the clonogenic assay (**Figure 12C**). The results suggest that GGH can inhibit the growth of cells in LUAD. The wound healing assay results, as shown in **Figure 12D**, demonstrated a reduced wound healing capability in LUAD cells following the silencing of GGH. The outcomes of transwell migration and invasion assays, depicted in **Figure 12E**, further indicated a decline in the migration and invasion abilities of the cells upon GGH silencing. Moreover, downregulation of GGH enhances the susceptibility of LUAD cells to ispinesib and epothilone-b (**Supplementary Figure 5**).

## Discussion

Cancer remains a leading cause of mortality worldwide (37). The prevalence and death rate of LC have been steadily rising annually (38). LUAD is the primary form of lung cancer, and its occurrence continues to be significant (3). The traditional TNM staging system, although fundamental in cancer diagnosis and management, exhibits considerable limitations in precisely assessing patient prognosis and informing treatment choices. This underscores the necessity for the discovery and implementation of new predictive tools to improve accuracy in prognosis and tailor treatment strategies more effectively (4). The COVID-19 outbreak is evolving into a significant worldwide issue concerning public health. New research indicates that CRGs may have crucial functions in viral infection, and they are also involved in the progression of numerous types of cancer. In LC cells, the study conducted by Kim et al. revealed that the suppression of MUC1-C signaling leads to a decrease in the levels of proteins associated with cell growth and an increase in the levels of proteins associated with cell death following SARS-CoV-2 infection (39). Additionally, a different study indicates that individuals with increased levels of ACE2, TMPRSS2, TLR1, TLR2, and TLR6 in LC are at a higher risk of contracting SARS CoV-2. This can result in a more severe SARS-CoV-2 infection and further contribute to the progression of cancer by activating NF-κB through TLR2 (40). The researches offer valuable starting points for further investigating the connection between COVID-19 and LUAD. This also motivated us to conduct additional bioinformatics analyses and study LUAD samples in order to gain a deeper understanding of the biological traits and clinical importance of CRGs in LUAD, as well as their potential relevance for LAUD treatments.

In this study, we aim to analyze the complete CRGs as comprehensively as possible. Therefore, we identified 34 DECRGs after collecting 322 CRGs from the HPA database, and subsequently found extensive genomic alterations in these DECRGs through multi-omics data analysis. Univariate cox analysis was conducted using these genes to identify potential prognostic CRGs for LUAD. Subsequently, we incorporated prognostic DECRGs into building a prognostic model for forecasting patient outcomes and performing a patient classification analysis. Based on the prognostic DECRGs expression profile of the TCGA training set, a total of 101 combinations were generated using 10 different machine learning algorithms. Additional assessment in three GEO testing sets demonstrated that the Cov-2S, which was the most suitable model, consisted of the combination of RFS. The Cov-2S demonstrated satisfactory performance in predicting OS in both TCGA and GEO datasets. The Cov-2S demonstrated superior clinical application potential compared to other clinical variables, as indicated by higher values of ROC AUC and C-index in various datasets. Interestingly, compared to the vast majority of previously reported prediction models, the predictive performance of Cov-2S remains superior. Additionally, additional examination indicated that Cov-2S acts as a separate determinant of the OS of patients with LUAD. Then, the median Cov-2S was utilized to classify both groups of participants into high and low Cov-2S groups. In both the TCGA training and GEO testing sets, it was observed that individuals with low Cov-2S had a longer survival time, suggesting that Cov-2S could serve as an unfavorable prognostic indicator.

“Hot” and “cold” tumors are an informal concept that represents the immunogenicity of tumors, with the former showing a high infiltration rate (41). On the contrary, “cold” tumors are distinguished by the absence or limited occurrence of lymphocytes in the TME, leading to a lack of response to ICIs therapy (42). Hence, recent research has focused on the potential and reality of transforming cold tumors into hot tumors, revealing the fluctuating alterations in the TME, with the aim of improving the effectiveness of ICIs treatment (43). Significantly, individuals with low Cov-2S exhibited elevated stromal and immune scores, along with reduced tumor purity in comparison to those with high Cov-2S. The interaction between immune cells and tumors was highly intricate, with distinct immune cells performing diverse functions. According to recent research, it has been demonstrated that Th2 cells possess the ability to promote tumors in LC and even in primary NSCLC tumors in humans (44). Current evidence indicates that B cells infiltrating tumors are involved in nearly every phase of LC (45). Cytotoxic cells, known as CD8+ T cells, stimulate antitumor responses by generating IFN-gamma (46). In line with the aforementioned discoveries, we observed that the low Cov-2S group exhibited increased levels of B cells and CD8+ T cells, as well as decreased levels of Th2 cells. These findings provide some insight into the improved OS of LUAD patients in the low Cov-2S group. Furthermore, the levels of immune checkpoints expression and the activities of the anti-tumor immunity cycles were notably increased in the low Cov-2S group. Several hallmarks associated with cancer, such as cell growth, genetic mending, and oxygen deprivation, exhibited greater activity in the high Cov-2S group. Further, this study found that the Cov-2S correlated with many immune-related and metabolic pathways. The collected evidence suggests that Cov-2S potentially plays a role in the progression of LUAD through the regulation of tumor immunity and metabolism. Additionally, patients with low Cov-2S exhibit signs of immune activity within the TME.

Earlier research suggested the comparison of tumors to “hot” and “cold” to explain their responsiveness to immunotherapy (41, 47). Tumors in this category, which have high TME scores, may be better suited for immunotherapy due to their increased presence of activated immunocytes and cells associated with inflammation. Hence, we hypothesized that individuals belonging to the low Cov-2S category might exhibit heightened responsiveness to immunotherapy, leading to extended periods of survival. In order to confirm our hypothesis, we will now examine how Cov-2S performs in terms of prognosis and its response to immunotherapy across different algorithms and datasets that have been treated with ICIs. TIDE has the potential to forecast the result of cancer patients who undergo treatment with initial anti-PD1 or anti-CTLA4 medications (31, 48). A higher TIDE score suggested a more favorable reaction to immunotherapy. The algorithm is utilized to assess the similarity in gene expression patterns between patients in distinct molecular subtypes and those with metastatic melanoma who received ICIs, facilitating submap analysis (32). The results of submap and TIDE analysis suggested that low Cov-2S was more promising for PD1 and CTLA4 treatment. The Cov-2S exhibited a significant decrease in patients who responded, as observed in the GSE78220, NIHMS1611472, and IMvigor210 datasets, in comparison to patients who did not respond. The performance of Cov-2S in prognosis and predicting immunotherapeutic response was indicated by all these indicators. Since chemotherapy is considered a crucial treatment for LUAD, we additionally examined the IC50 value of typical medications for each LUAD patients. Consequently, LUAD patients with high Cov-2S exhibited lower IC50 values for paclitaxel, SB-743921, ispinesib, epothilone-b, and cabazitaxel, indicating a potential higher susceptibility to chemotherapy in these individuals. Hence, implementing various approaches could enhance the clinical result for individuals. For example, tumors that were considered “hot” were advised to undergo therapy specifically targeting T-cells (49, 50). The sensitivity of “cold” tumors can be improved by combining chemotherapy with T cell enhancement or stimulatory signals (51). Considering the TME score when selecting LUAD treatment could enhance the survival outcome of patients.

In our research, we developed a Cov-2S signature composed of 10 CRGs, which emerged as a promising prognostic predictor for LUAD. Notably, the majority of these genes have been previously reported to be involved in the onset and progression of LC, underscoring their relevance in the disease’s pathology. PKP2 is a member of the plaque-bound plakophilins family and is extensively present in epithelial cells (52). PKP2 is overexpressed in LUAD tissues and its high expression correlates with poor outcome of patients. PKP2 overexpression enhances proliferation and invasion of LUAD cells, and this effect is potentiated by EGFR activation (53). Cheng et al. discovered that inhibiting PKP2 methylation reduces its binding affinity to β-catenin, thereby overcoming the radioresistance in LUAD cells (54). CENPF functions as a component of the centromere-kinetochore complex and as an element of the nuclear matrix during G2 phase of interphase (55). CENPF was overexpressed in LUAD tissues and cell lines (56). The progression of LUAD is influenced by both CENPF and ERβ2/5, and inhibiting the expression of ER2/5 can impede the advancement of LUAD through the knockdown of CENPF (57). CHPF, a 775 amino acid type II transmembrane protein belonging to the chondroitin synthase family, was found overexpressed in LC tissues, correlating with reduced OS (58). *In vitro* experiments revealed that CHPF stimulated the proliferation, migration, and invasion of LC cells, as well as induced tumorigenesis *in vivo* (59). FKBP10, an endoplasmic reticulum chaperone with four PPIase domains, exhibits an expression inversely related to the survival of LC patients (60). Through its PPIase activity, FKBP10 mechanistically enhances both cancer growth and stemness. Also, FKBP10 is involved in interactions with ribosomes. The downregulation of FKBP10 has been shown to lead to a reduction in translation elongation, particularly at the start of open reading frames (61). FBLN5, a newly discovered member of the fibulin family, has the ability to inhibit angiogenesis in a manner that relies on the presence of RGD (62). Activation of the ERK pathway induces MMP-7 expression, facilitating LC invasion and metastasis, by promoting epigenetic suppression of FBLN5 (63). F2RL1 belongs to the extensively researched G protein-coupled receptor family, recognized as significant targets in drug development (64). The inhibition of F2RL1 significantly increased the effectiveness of gefitinib in regulating EGFR transactivation, cell survival, movement, and programmed cell death in LC cells (65). Moreover, inhibiting F2RL1 could restrict ERK-induced epithelial-mesenchymal transition (EMT) and immune checkpoints, thereby reducing EGFR transactivation and reactivating osimertinib (66). We conducted qRT-PCR to evaluate the expression of 10 CRGs in LUAD cells, ultimately selecting GGH for experimental validation due to its markedly differential expression and lack of prior reporting in LUAD. IHC assay further confirmed high expression of GGH protein in LUAD tissues. The functional trials revealed that the robust inhibition of LUAD cell growth, proliferation, migration, and invasion was observed when siRNA induced GGH silencing, indicating a potential oncogenic function of GGH in LUAD.

Apart from the encouraging results of this work, we were also aware of the limitations of the present study. Although our research has the merit of using large cohorts from multiple databases for the generation and verification of the Cov-2S, the present study is still retrospective in nature. There is a need for a prospective set study to further validate the utility of the Cov-2S. We conducted preliminary validation of the expression levels and biological functions of key genes in the Cov-2S only in LUAD cells and tissues, further validation in clinical samples and further mechanistic investigation are needed.

## Conclusion

In conclusion, the Cov-2S identified in this research is an innovative prognostic indicator that reveals fresh targets for therapy and new theoretical principles for assessing the prognosis and personalized treatment of LUAD. Furthermore, our experiments validated that GGH functioned as a cancer-causing gene that could potentially enhance the advancement of LUAD.

## Data availability statement

The datasets presented in this study can be found in online repositories. The names of the repository/repositories and accession number(s) can be found in the article/**Supplementary Material**.

## Ethics statement

The studies involving humans were approved by ethics committee and institutional review board of the Outdo Biotech. Co., Ltd. (SHYJS-CP-1804018). The studies were conducted in accordance with the local legislation and institutional requirements. The participants provided their written informed consent to participate in this study.

## Author contributions

YW: Formal analysis, Investigation, Writing – original draft. YX: Writing – original draft, Formal analysis, Validation, Writing – review & editing, Supervision. YD: Formal analysis, Investigation, Writing – original draft. LY: Resources, Writing – review & editing. DW: Visualization, Writing – review & editing. ZY: Writing – review & editing. YZ: Conceptualization, Project administration, Supervision, Writing – review & editing.

## References

[1] Bray F Ferlay J Soerjomataram I Siegel RL Torre LA Jemal A. Global cancer statistics 2018: GLOBOCAN estimates of incidence and mortality worldwide for 36 cancers in 185 countries. CA Cancer J Clin. (2018) 68:394–424. doi: 10.3322/caac.21492 30207593

[2] Basu A Bodycombe NE Cheah JH Price EV Liu K Schaefer GI. An interactive resource to identify cancer genetic and lineage dependencies targeted by small molecules. Cell. (2013) 154:1151–61. doi: 10.1016/j.cell.2013.08.003 PMC3954635 23993102

[3] Siegel RL Miller KD Wagle NS Jemal A. Cancer statistics, 2023. CA Cancer J Clin. (2023) 73:17–48. doi: 10.3322/caac.21763 36633525

[4] Thai AA Solomon BJ Sequist LV Gainor JF Heist RS. Lung cancer. Lancet. (2021) 398:535–54. doi: 10.1016/s0140-6736(21)00312-3 34273294

[5] Duma N Santana-Davila R Molina JR. Non-small cell lung cancer: epidemiology, screening, diagnosis, and treatment. Mayo Clin Proc. (2019) 94:1623–40. doi: 10.1016/j.mayocp.2019.01.013 31378236

[6] Sholl LM. Biomarkers in lung adenocarcinoma: a decade of progress. Arch Pathol Lab Med. (2015) 139:469–80. doi: 10.5858/arpa.2014-0128-RA 25255293

[7] Tavernari D Battistello E Dheilly E Petruzzella AS Mina M Sordet-Dessimoz J. Nongenetic evolution drives lung adenocarcinoma spatial heterogeneity and progression. Cancer Discovery. (2021) 11:1490–507. doi: 10.1158/2159-8290.Cd-20-1274 33563664

[8] Zhang C Zhang J Xu FP Wang YG Xie Z Su J. Genomic landscape and immune microenvironment features of preinvasive and early invasive lung adenocarcinoma. J Thorac Oncol. (2019) 14:1912–23. doi: 10.1016/j.jtho.2019.07.031 PMC6986039 31446140

[9] Safiabadi Tali SH LeBlanc JJ Sadiq Z Oyewunmi OD Camargo C Nikpour B. Tools and techniques for severe acute respiratory syndrome coronavirus 2 (SARS-coV-2)/COVID-19 detection. Clin Microbiol Rev. (2021) 34(3):e00228-20. doi: 10.1128/cmr.00228-20 33980687 PMC8142517

[10] Dai M Liu D Liu M Zhou F Li G Chen Z. Patients with cancer appear more vulnerable to SARS-coV-2: A multicenter study during the COVID-19 outbreak. Cancer Discovery. (2020) 10:783–91. doi: 10.1158/2159-8290.Cd-20-0422 PMC7309152 32345594

[11] Liang W Guan W Chen R Wang W Li J Xu K. Cancer patients in SARS-CoV-2 infection: a nationwide analysis in China. Lancet Oncol. (2020) 21:335–7. doi: 10.1016/s1470-2045(20)30096-6 PMC7159000 32066541

[12] White MK Pagano JS Khalili K. Viruses and human cancers: a long road of discovery of molecular paradigms. Clin Microbiol Rev. (2014) 27:463–81. doi: 10.1128/cmr.00124-13 PMC4135891 24982317

[13] Müller-Coan BG Caetano BFR Pagano JS Elgui de Oliveira D. Cancer progression goes viral: the role of oncoviruses in aggressiveness of Malignancies. Trends Cancer. (2018) 4:485–98. doi: 10.1016/j.trecan.2018.04.006 29937047

[14] Mesri EA Feitelson MA Munger K. Human viral oncogenesis: a cancer hallmarks analysis. Cell Host Microbe. (2014) 15:266–82. doi: 10.1016/j.chom.2014.02.011 PMC3992243 24629334

[15] Krump NA You J. Molecular mechanisms of viral oncogenesis in humans. Nat Rev Microbiol. (2018) 16:684–98. doi: 10.1038/s41579-018-0064-6 PMC6336458 30143749

[16] Su S Wong G Shi W Liu J Lai ACK Zhou J. Epidemiology, genetic recombination, and pathogenesis of coronaviruses. Trends Microbiol. (2016) 24:490–502. doi: 10.1016/j.tim.2016.03.003 27012512 PMC7125511

[17] Geisslinger F Vollmar AM Bartel K. Cancer patients have a higher risk regarding COVID-19 - and vice versa? Pharm (Basel). (2020) 13(7):143. doi: 10.3390/ph13070143 PMC7408191 32640723

[18] Song D Jia X Liu X Hu L Lin K Xiao T. Identification of the receptor of oncolytic virus M1 as a therapeutic predictor for multiple solid tumors. Signal Transduct Target Ther. (2022) 7:100. doi: 10.1038/s41392-022-00921-3 35393389 PMC8989880

[19] Jiang F Lu DF Zhan Z Yuan GQ Liu GJ Gu JY. SARS-coV-2 pattern provides a new scoring system and predicts the prognosis and immune therapeutic response in glioma. Cells. (2022) 11(24):3997. doi: 10.3390/cells11243997 36552760 PMC9777143

[20] Wang Z Yuan Q Chen X Luo F Shi X Guo F. A prospective prognostic signature for pancreatic adenocarcinoma based on ubiquitination-related mRNA-lncRNA with experimental validation in *vitro* and vivo. Funct Integr Genomics. (2023) 23:263. doi: 10.1007/s10142-023-01158-1 37540295 PMC10403435

[21] Subramanian A Tamayo P Mootha VK Mukherjee S Ebert BL Gillette MA. Gene set enrichment analysis: a knowledge-based approach for interpreting genome-wide expression profiles. Proc Natl Acad Sci USA. (2005) 102:15545–50. doi: 10.1073/pnas.0506580102 PMC1239896 16199517

[22] Liu Z Liu L Weng S Guo C Dang Q Xu H. Machine learning-based integration develops an immune-derived lncRNA signature for improving outcomes in colorectal cancer. Nat Commun. (2022) 13:816. doi: 10.1038/s41467-022-28421-6 35145098 PMC8831564

[23] Heagerty PJ Lumley T Pepe MS. Time-dependent ROC curves for censored survival data and a diagnostic marker. Biometrics. (2000) 56:337–44. doi: 10.1111/j.0006-341X.2000.00337.x 10877287

[24] Wilkerson MD Hayes DN. ConsensusClusterPlus: a class discovery tool with confidence assessments and item tracking. Bioinformatics. (2010) 26:1572–3. doi: 10.1093/bioinformatics/btq170 PMC2881355 20427518

[25] Reich M Liefeld T Gould J Lerner J Tamayo P Mesirov JP. GenePattern 2.0. Nat Genet. (2006) 38:500–1. doi: 10.1038/ng0506-500 16642009

[26] Xu L Deng C Pang B Zhang X Liu W Liao G. TIP: A web server for resolving tumor immunophenotype profiling. Cancer Res. (2018) 78:6575–80. doi: 10.1158/0008-5472.Can-18-0689 30154154

[27] Yoshihara K Shahmoradgoli M Martínez E Vegesna R Kim H Torres-Garcia W. Inferring tumour purity and stromal and immune cell admixture from expression data. Nat Commun. (2013) 4:2612. doi: 10.1038/ncomms3612 24113773 PMC3826632

[28] Li T Fan J Wang B Traugh N Chen Q Liu JS. TIMER: A web server for comprehensive analysis of tumor-infiltrating immune cells. Cancer Res. (2017) 77:e108–10. doi: 10.1158/0008-5472.Can-17-0307 PMC6042652 29092952

[29] Becht E Giraldo NA Lacroix L Buttard B Elarouci N Petitprez F. Estimating the population abundance of tissue-infiltrating immune and stromal cell populations using gene expression. Genome Biol. (2016) 17:218. doi: 10.1186/s13059-016-1070-5 27765066 PMC5073889

[30] Yi M Nissley DV McCormick F Stephens RM. ssGSEA score-based Ras dependency indexes derived from gene expression data reveal potential Ras addiction mechanisms with possible clinical implications. Sci Rep. (2020) 10:10258. doi: 10.1038/s41598-020-66986-8 32581224 PMC7314760

[31] Jiang P Gu S Pan D Fu J Sahu A Hu X. Signatures of T cell dysfunction and exclusion predict cancer immunotherapy response. Nat Med. (2018) 24:1550–8. doi: 10.1038/s41591-018-0136-1 PMC6487502 30127393

[32] Hoshida Y Brunet JP Tamayo P Golub TR Mesirov JP. Subclass mapping: identifying common subtypes in independent disease data sets. PloS One. (2007) 2:e1195. doi: 10.1371/journal.pone.0001195 18030330 PMC2065909

[33] Seashore-Ludlow B Rees MG Cheah JH Cokol M Price EV Coletti ME. Harnessing connectivity in a large-scale small-molecule sensitivity dataset. Cancer Discovery. (2015) 5:1210–23. doi: 10.1158/2159-8290.Cd-15-0235 PMC4631646 26482930

[34] Anghel CV Quon G Haider S Nguyen F Deshwar AG Morris QD. ISOpureR: an R implementation of a computational purification algorithm of mixed tumour profiles. BMC Bioinf. (2015) 16:156. doi: 10.1186/s12859-015-0597-x PMC4429941 25972088

[35] Geeleher P Cox N Huang RS. pRRophetic: an R package for prediction of clinical chemotherapeutic response from tumor gene expression levels. PloS One. (2014) 9:e107468. doi: 10.1371/journal.pone.0107468 25229481 PMC4167990

[36] Zhang Y Wang Y Chen J Xia Y Huang Y. A programmed cell death-related model based on machine learning for predicting prognosis and immunotherapy responses in patients with lung adenocarcinoma. Front Immunol. (2023) 14:1183230. doi: 10.3389/fimmu.2023.1183230 37671155 PMC10475728

[37] Liu S Tian W Ma Y Li J Yang J Li B. Serum exosomal proteomics analysis of lung adenocarcinoma to discover new tumor markers. BMC Cancer. (2022) 22:279. doi: 10.1186/s12885-022-09366-x 35291954 PMC8925168

[38] Bade BC Dela Cruz CS. Lung cancer 2020: epidemiology, etiology, and prevention. Clin Chest Med. (2020) 41:1–24. doi: 10.1016/j.ccm.2019.10.001 32008623

[39] Kim D Maharjan S Kim J Park S Park JA Park BK. MUC1-C influences cell survival in lung adenocarcinoma Calu-3 cells after SARS-CoV-2 infection. BMB Rep. (2021) 54:425–30. doi: 10.5483/BMBRep.2021.54.8.018 PMC8411043 33832550

[40] Kim MJ Kim JY Shin JH Son J Kang Y Jeong SK. The SARS-CoV-2 spike protein induces lung cancer migration and invasion in a TLR2-dependent manner. Cancer Commun (Lond). (2023) 44(2):273–277. doi: 10.1002/cac2.12485 PMC10876188 37702496

[41] Galon J Bruni D. Approaches to treat immune hot, altered and cold tumours with combination immunotherapies. Nat Rev Drug Discovery. (2019) 18:197–218. doi: 10.1038/s41573-018-0007-y 30610226

[42] Ochoa de Olza M Navarro Rodrigo B Zimmermann S Coukos G. Turning up the heat on non-immunoreactive tumours: opportunities for clinical development. Lancet Oncol. (2020) 21:e419–30. doi: 10.1016/s1470-2045(20)30234-5 32888471

[43] Hu R Han Q Zhang J. STAT3: A key signaling molecule for converting cold to hot tumors. Cancer Lett. (2020) 489:29–40. doi: 10.1016/j.canlet.2020.05.035 32522692

[44] Zhong R Zhang Y Chen D Cao S Han B Zhong H. Single-cell RNA sequencing reveals cellular and molecular immune profile in a Pembrolizumab-responsive PD-L1-negative lung cancer patient. Cancer Immunol Immunother. (2021) 70:2261–74. doi: 10.1007/s00262-021-02848-0 PMC10991356 33506299

[45] Wang SS Liu W Ly D Xu H Qu L Zhang L. Tumor-infiltrating B cells: their role and application in anti-tumor immunity in lung cancer. Cell Mol Immunol. (2019) 16:6–18. doi: 10.1038/s41423-018-0027-x 29628498 PMC6318290

[46] St Paul M Ohashi PS. The roles of CD8(+) T cell subsets in antitumor immunity. Trends Cell Biol. (2020) 30:695–704. doi: 10.1016/j.tcb.2020.06.003 32624246

[47] Galon J Costes A Sanchez-Cabo F Kirilovsky A Mlecnik B Lagorce-Pagès C. Type, density, and location of immune cells within human colorectal tumors predict clinical outcome. Science. (2006) 313:1960–4. doi: 10.1126/science.1129139 17008531

[48] Fu J Li K Zhang W Wan C Zhang J Jiang P. Large-scale public data reuse to model immunotherapy response and resistance. Genome Med. (2020) 12:21. doi: 10.1186/s13073-020-0721-z 32102694 PMC7045518

[49] Buchbinder EI Desai A. CTLA-4 and PD-1 pathways: similarities, differences, and implications of their inhibition. Am J Clin Oncol. (2016) 39:98–106. doi: 10.1097/coc.0000000000000239 26558876 PMC4892769

[50] Hellmann MD Friedman CF Wolchok JD. Combinatorial cancer immunotherapies. Adv Immunol. (2016) 130:251–77. doi: 10.1016/bs.ai.2015.12.005 26923003

[51] Whiteside TL Demaria S Rodriguez-Ruiz ME Zarour HM Melero I. Emerging opportunities and challenges in cancer immunotherapy. Clin Cancer Res. (2016) 22:1845–55. doi: 10.1158/1078-0432.Ccr-16-0049 PMC4943317 27084738

[52] Bass-Zubek AE Godsel LM Delmar M Green KJ. Plakophilins: multifunctional scaffolds for adhesion and signaling. Curr Opin Cell Biol. (2009) 21:708–16. doi: 10.1016/j.ceb.2009.07.002 PMC3091506 19674883

[53] Hao XL Tian Z Han F Chen JP Gao LY Liu JY. Plakophilin-2 accelerates cell proliferation and migration through activating EGFR signaling in lung adenocarcinoma. Pathol Res Pract. (2019) 215:152438. doi: 10.1016/j.prp.2019.152438 31126818

[54] Cheng C Pei X Li SW Yang J Li C Tang J. CRISPR/Cas9 library screening uncovered methylated PKP2 as a critical driver of lung cancer radioresistance by stabilizing β-catenin. Oncogene. (2021) 40:2842–57. doi: 10.1038/s41388-021-01692-x 33742119

[55] Liao H Winkfein RJ Mack G Rattner JB Yen TJ. CENP-F is a protein of the nuclear matrix that assembles onto kinetochores at late G2 and is rapidly degraded after mitosis. J Cell Biol. (1995) 130:507–18. doi: 10.1083/jcb.130.3.507 PMC2120529 7542657

[56] Li MX Zhang MY Dong HH Li AJ Teng HF Liu AL. Overexpression of CENPF is associated with progression and poor prognosis of lung adenocarcinoma. Int J Med Sci. (2021) 18:494–504. doi: 10.7150/ijms.49041 33390818 PMC7757141

[57] Hexiao T Yuquan B Lecai X Yanhong W Li S Weidong H. Knockdown of CENPF inhibits the progression of lung adenocarcinoma mediated by ERβ2/5 pathway. Aging (Albany NY). (2021) 13:2604–25. doi: 10.18632/aging.202303 PMC7880349 33428600

[58] Siebert JR Conta Steencken A Osterhout DJ. Chondroitin sulfate proteoglycans in the nervous system: inhibitors to repair. BioMed Res Int. (2014) 2014:845323. doi: 10.1155/2014/845323 25309928 PMC4182688

[59] Cao C Liu Y Wang Q Zhao J Shi M Zheng J. Expression of CHPF modulates cell proliferation and invasion in lung cancer. Braz J Med Biol Res. (2020) 53:e9021. doi: 10.1590/1414-431x20209021 32348423 PMC7205412

[60] Chen Y Terajima M Banerjee P Guo H Liu X Yu J. FKBP65-dependent peptidyl-prolyl isomerase activity potentiates the lysyl hydroxylase 2-driven collagen cross-link switch. Sci Rep. (2017) 7:46021. doi: 10.1038/srep46021 28378777 PMC5380960

[61] Ramadori G Ioris RM Villanyi Z Firnkes R Panasenko OO Allen G. FKBP10 regulates protein translation to sustain lung cancer growth. Cell Rep. (2020) 30:3851–3863.e6. doi: 10.1016/j.celrep.2020.02.082 32187554

[62] Albig AR Neil JR Schiemann WP. Fibulins 3 and 5 antagonize tumor angiogenesis in *vivo* . Cancer Res. (2006) 66:2621–9. doi: 10.1158/0008-5472.Can-04-4096 16510581

[63] Yue W Sun Q Landreneau R Wu C Siegfried JM Yu J. Fibulin-5 suppresses lung cancer invasion by inhibiting matrix metalloproteinase-7 expression. Cancer Res. (2009) 69:6339–46. doi: 10.1158/0008-5472.Can-09-0398 PMC2719681 19584278

[64] Pawar NR Buzza MS Antalis TM. Membrane-anchored serine proteases and protease-activated receptor-2-mediated signaling: co-conspirators in cancer progression. Cancer Res. (2019) 79:301–10. doi: 10.1158/0008-5472.Can-18-1745 PMC6335149 30610085

[65] Jiang Y Zhuo X Fu X Wu Y Mao C. Targeting PAR2 overcomes gefitinib resistance in non-small-cell lung cancer cells through inhibition of EGFR transactivation. Front Pharmacol. (2021) 12:625289. doi: 10.3389/fphar.2021.625289 33967759 PMC8100583

[66] Jiang Y Zhuo X Wu Y Fu X Mao C. PAR2 blockade reverses osimertinib resistance in non-small-cell lung cancer cells via attenuating ERK-mediated EMT and PD-L1 expression. Biochim Biophys Acta Mol Cell Res. (2022) 1869:119144. doi: 10.1016/j.bbamcr.2021.119144 34599981

