# Supplementary material for: Computational identification and experimental verification of a novel signature based on SARS-CoV-2–related genes for predicting prognosis, immune microenvironment and therapeutic strategies in lung adenocarcinoma patients
Source: Front Immunol. 2024 Mar 26;15:1366928. doi: 10.3389/fimmu.2024.1366928 (PMC11004994; doi:10.3389/fimmu.2024.1366928)
Supplement: Supplementary file 2 [file DataSheet_1.pdf]

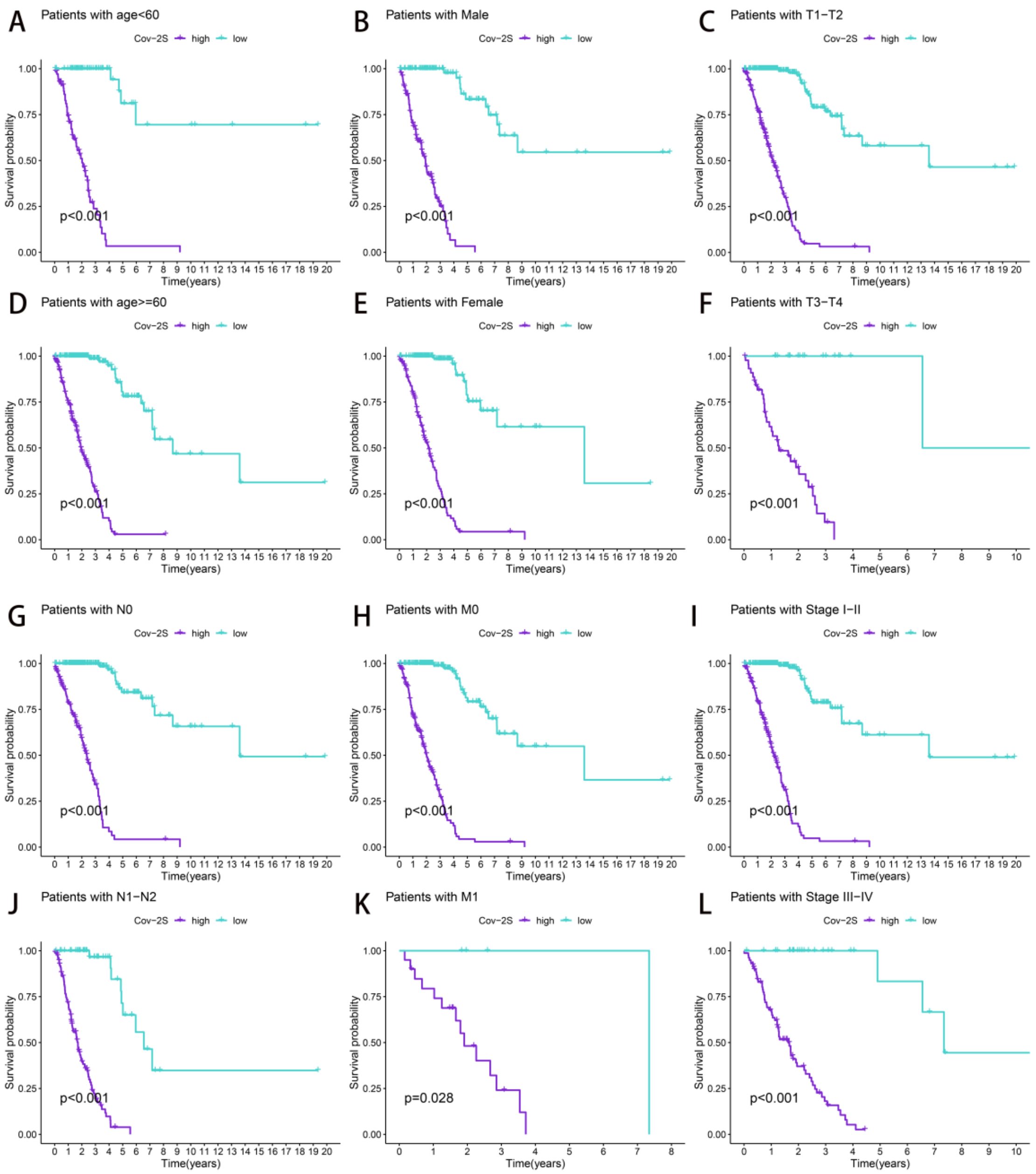


**Supplementary Figure 1 The survival curves of the Cov-2S stratified by age, gender, T, N, M and stage. (A)** ≥60 years, **(B)** male, **(C)** T1-2, **(D)** <60 years, **(E)** female, **(F)** T3-4, **(G)** N0, **(H)** M0, **(I)** stage1-2, **(J)** N1-2, **(K)** M1, **(L)** stage3-4.

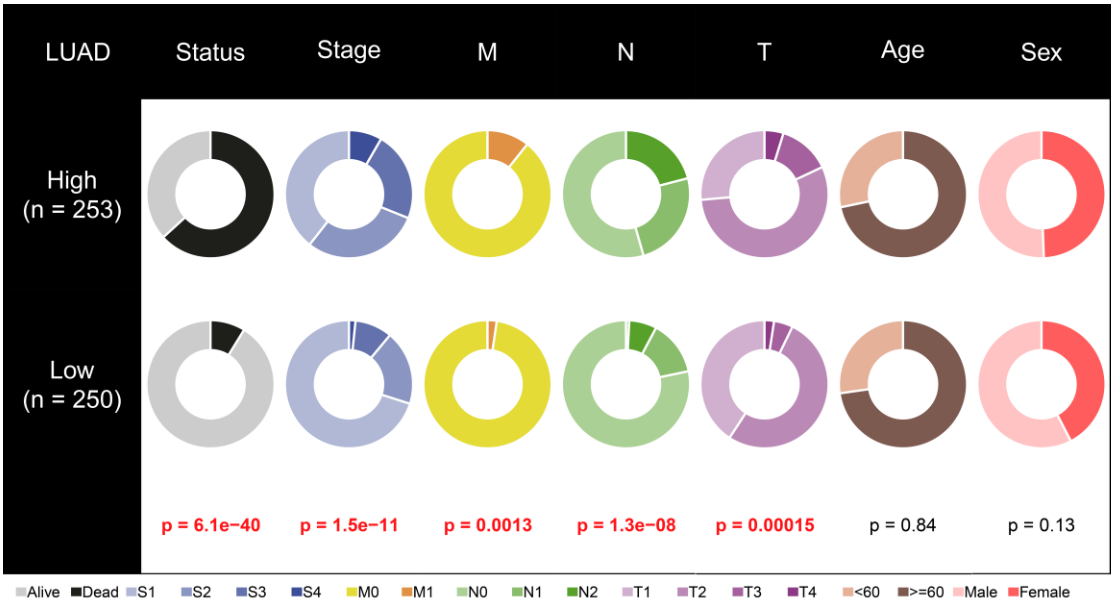


**Supplementary Figure 2 The circular pie chart for the proportion difference of clinical indices.**

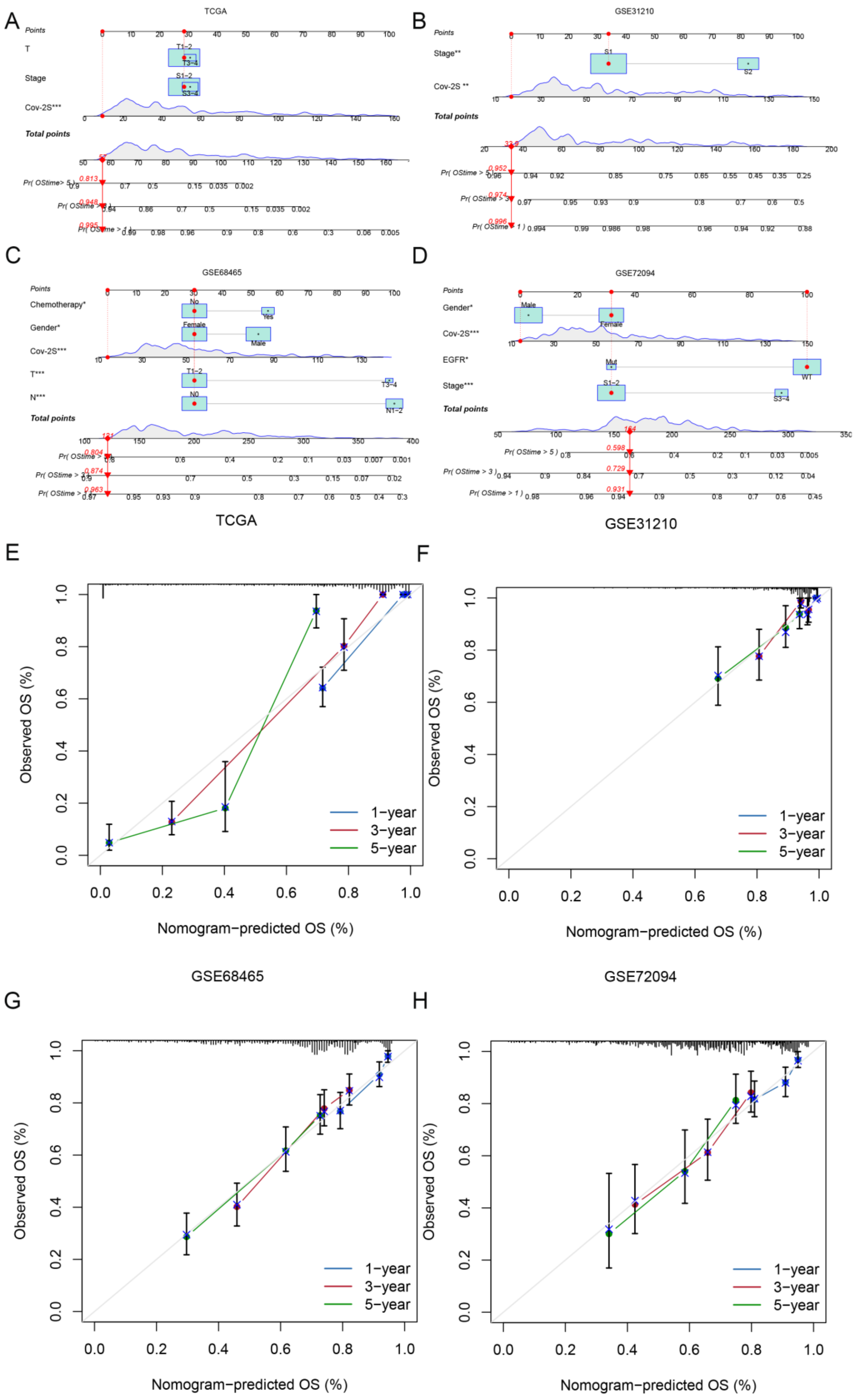
A
TCGA
Points
T
Stage
Cov-2S***
Total points
B
GSE31210
Points
Stage**
Cov-2S **
Total points
C
GSE68465
Points
Chemotherapy*
Gender*
Cov-2S***
T***
N***
Total points
D
GSE72094
Points
Gender*
Cov-2S***
EGFR*
Stage***
Total points
TCGA
E
GSE31210
F
GSE68465
G
GSE72094
H
Observed OS (%)
Nomogram−predicted OS (%)
1−year
3−year
5−year

**Supplementary Figure 3 Nomogram construction and evaluation. (A-D)** Nomogram model for the independent predictors in TCGA, GSE31210, GSE68465 and GSE72094 datasets. **(E-H)** Calibration curve for nomogram in TCGA, GSE31210, GSE68465 and GSE72094 datasets..

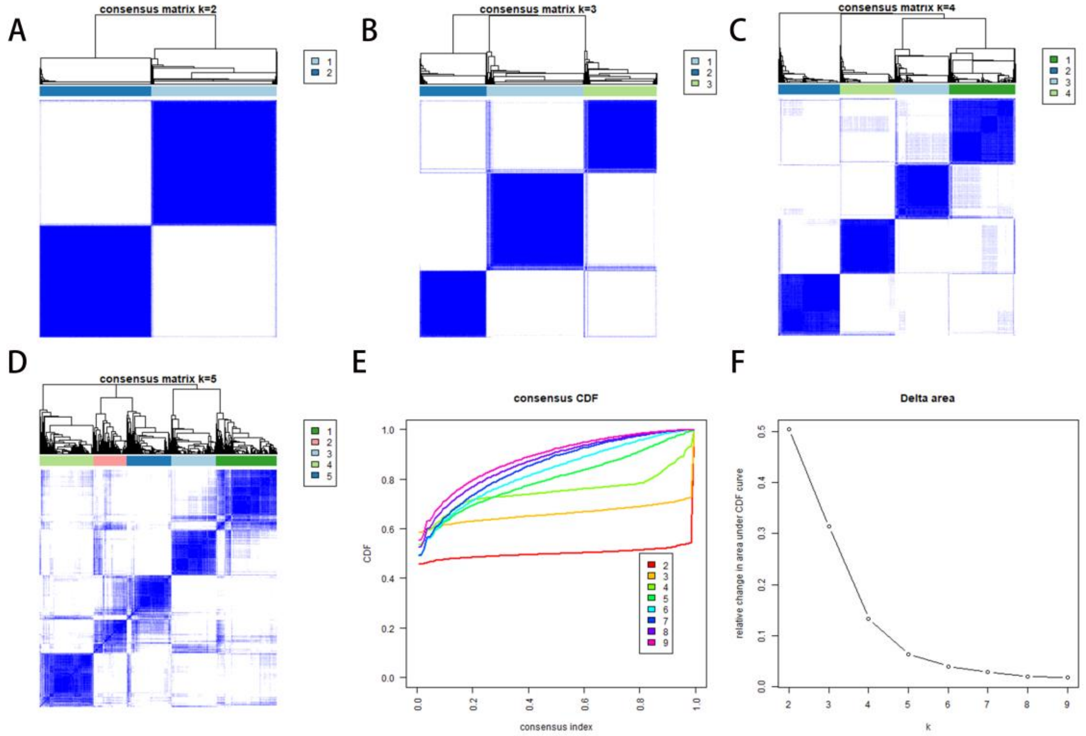


**Supplementary Figure 4 Consensus matrixes of all LUAD patients based on Cov-2S. (A-D)** Consensus matrixes of all LUAD patients for each k (k = 2–5). **(E)** Cumulative distribution function curves for unsupervised clustering of LUAD based on Cov-2S, k = 2-9. **(F)** Relative change in area under the CDF curve for unsupervised clustering of LUAD, k =2-9.

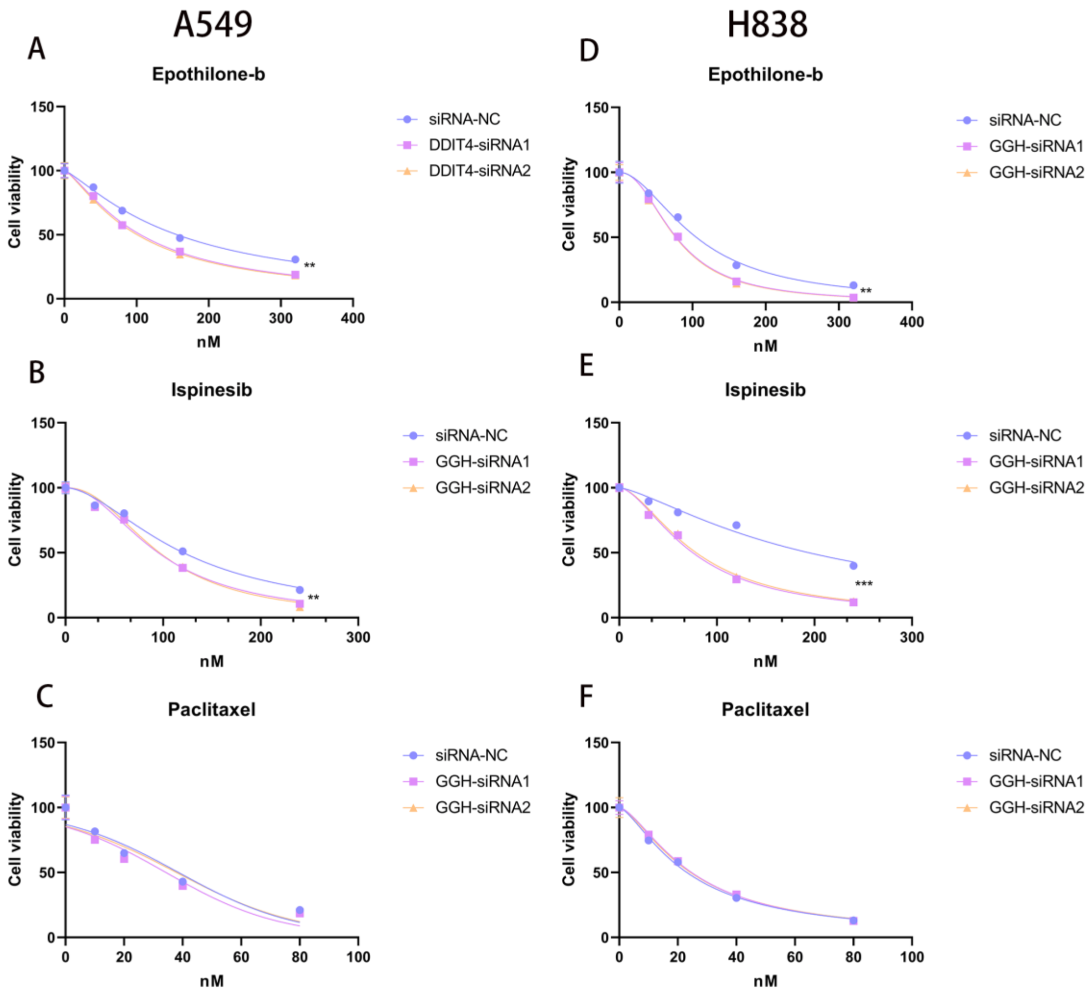


**Supplementary Figure 5 Effect of GGH on chemotherapy sensitivity of LUAD cells.**

**(A-C)** Effect of GGH knockdown on epothilone-b, ispinesib and paclitaxel of A549 cell.

**(D-F)** Effect of GGH knockdown on epothilone-b, ispinesib and paclitaxel of H838 cell.
